# Archaeal type six secretion system mediates contact-dependent antagonism

**DOI:** 10.1126/sciadv.adp7088

**Published:** 2024-11-15

**Authors:** Tobias Zachs, Jessie James L. Malit, Jingwei Xu, Alexandra Schürch, Shamphavi Sivabalasarma, Phillip Nußbaum, Sonja-Verena Albers, Martin Pilhofer

**Affiliations:** ^1^ Department of Biology, Institute of Molecular Biology & Biophysics, Eidgenössische Technische Hochschule Zürich, Otto-Stern-Weg 5, 8093 Zürich, Switzerland.; ^2^ Molecular Biology of Archaea, Institute of Biology, Faculty of Biology, University of Freiburg, Schänzlestr. 1, 79104 Freiburg, Germany.; ^3^ Spemann Graduate School of Biology and Medicine, University of Freiburg, 79104 Freiburg, Germany.

## Abstract

Microbial communities are shaped by cell-cell interactions. Although archaea are often found in associations with other microorganisms, the mechanisms structuring these communities are poorly understood. Here, we report on the structure and function of haloarchaeal contractile injection systems (CISs). Using a combination of functional assays and time-lapse imaging, we show that *Halogeometricum borinquense* exhibits antagonism toward *Haloferax volcanii* by inducing cell lysis and inhibiting proliferation. This antagonism is contact-dependent and requires a functional CIS, which is encoded by a gene cluster that is associated with toxin-immunity pairs. Cryo–focused ion beam milling and imaging by cryo–electron tomography revealed that these CISs are bound to the cytoplasmic membrane, resembling the bacterial type six secretion systems (T6SSs). We show that related T6SS gene clusters are conserved and expressed in other haloarchaeal strains, which exhibit antagonistic behavior. Our data provide a mechanistic framework for understanding how archaea may shape microbial communities and affect the food webs they inhabit.

## Teaser

Haloarchaea use contractile injection systems to facilitate contact-dependent antagonism against other haloarchaea.

## INTRODUCTION

Ecological niches frequently contain a complex, yet defined, mixture of different microorganisms (*1*, *2*). Bacteria use a wide range of highly adapted secretion systems to translocate arsenals of toxins across their cell envelopes (*3*). This allows them to obtain a competitive advantage by structuring their surrounding community and also grants them access to alternative substrate sources. Archaea are also known to exist in complex microbiomes, including biofilms and microbial consortia (*2*, *4*); however, studies of these communities have primarily focused on the metabolic flux between different members (*5*–*8*). With the exception of archaeocins, mechanistic insights into how archaea obtain a competitive advantage and use antagonism to define an archaeal niche are largely lacking (*9*–*11*).

Recent bioinformatics studies revealed the presence of contractile injection system (CIS) gene clusters in archaeal genomes from different phyla (*11*–*14*). Related CIS gene clusters are widespread among diverse bacteria and are known to mediate a wide range of interactions between bacteria and other organisms (*3*). With a structure and mechanism that is evolutionarily related to contractile phages, like the T4 phage, CISs allow the host to translocate effector proteins into the surrounding environment or directly into neighboring cells (*15*–*17*). CISs have adapted to a range of functions, but their conserved core is composed of a sheath-tube module (also called a contractile tail), which assembles onto a baseplate. To facilitate toxin secretion, the baseplate triggers contraction of a dynamic outer sheath forcing a rigid inner tube, tipped with a spike protein, to be driven through membranes (*18*). This dynamic transition from a primed extended state to a fired contracted state is a hallmark of CISs. The process by which this occurs—i.e., their mode of action—can vary.

The two most commonly studied modes of action are the “type six secretion systems” (T6SSs) and the “extracellular CISs” (eCISs). T6SSs assemble and fire while attached to the host’s cytoplasmic membrane. To date, T6SSs have only been described in Gram-negative bacteria, which use these systems to translocate cargo across the cell envelope in a cell-cell contact-dependent manner (*19*, *20*). eCISs, in contrast, are assembled within the bacterial host cell but are only functional when released into the environment by cell lysis. Once in contact with a target cell, eCISs use their tail fibers to bind and subsequently contract to inject their cargo (*21*–*24*).

The bioinformatics identification of CIS gene clusters in archaea raises pressing questions regarding their expression, mode of action, and function, offering exciting prospects for understanding how archaea may structure the communities that they thrive in.

## RESULTS

### *H. borinquense* assembles contractile sheaths composed of Cis2

**Fig. 1.**
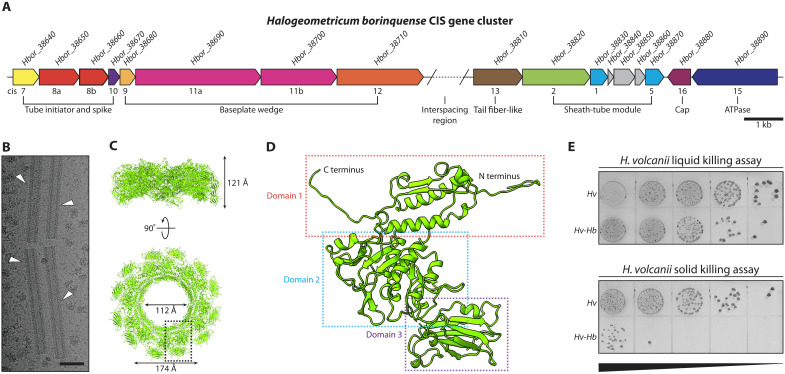
*H. borinquense* expresses a CIS and exhibits antagonism. (**A**) Schematic of the *H. borinquense* CIS gene cluster found on the plasmid pHBOR03. Annotations include the gene accession numbers (*Hbor_38640-38890*) and the CIS gene nomenclature (*cis1-16*) with genes grouped together by predicted functional groups. For details on the interspacing region, see Fig. 4. ATPase, adenosine triphosphatase. (**B**) Micrograph of plunge frozen sheaths isolated from *H. borinquense*. Micrograph shows sheath structures used for single-particle helical reconstruction (white arrowheads). Sheath lengths were heterogeneous. Scale bar, 50 nm. (**C**) Side and top views of four layers of the contracted sheath structure composed of Cis2 with corresponding dimensions. Single subunit within the dashed box is depicted in (D). (**D**) Structure of the contracted sheath monomer derived from a single-particle helical reconstruction at a resolution of 2.9 Å. Within the sheath monomer, three domains are observable, with only a section between domains 2 and 3 being unresolved due to flexibility (residues 209 to 291). (**E**) Killing assay performed by coincubating *H. borinquense* (*Hb*) with *H. volcanii* (*Hv*) in liquid and on solid media for 24 hours at 45°C. Shown are serial dilution spots (10^−2^ to 10^−6^) spotted on an *H. volcanii* selection plate. Note the substantial drop (1000-fold) in the growth of *H. volcanii* at higher dilutions in the solid killing assay.

We chose to establish *Halogeometricum borinquense* (hereafter *H. borinquense* or *Hb*) as the model system for investigating archaeal CIS gene clusters. *Hb* is a halophilic archaeon that was originally isolated from the solar salterns of Cabo Rojo, Puerto Rico (*25*, *26*). Its genome was reported to have genes with similarities to a diverse group of related CISs (*13*). These CIS gene clusters are phylogenetically part of “clade IIa,” which, to date, has no characterized representatives. We reanalyzed the *Hb* CIS gene cluster (accessions: *Hbor_38640-38890*), which is encoded on a 210-kb genomic plasmid (pHBOR03) (Fig. 1A). Fourteen genes with similarities to previously identified structural components of the bacterial CISs are present in the gene cluster. These structural genes are organized in two subclusters, which are separated by an ~8.7-kb “interspacing” region, encoding for nine hypothetical protein genes. The 14 structural genes were renamed according to their homologies with previously characterized components of the bacterial CISs (table S1) (*24*, *27*). All but homologs for the plug (*cis6*), tape measure (*cis14*), and crown (*cis19*) were found in the gene cluster.

Next, we tested for the expression of *cis* genes by performing crude purifications of CISs from *Hb* cultures. Electron microscopy (EM) imaging revealed an abundance of sheath-like structures that were always in a contracted state with a mean length of 220 ± 16 nm (Fig. 1B and fig. S1, A and B). Mass spectrometry analysis of these preparations detected the presence of *Hb* protein Cis2 (*Hbor_38820*), but no other CIS gene cluster components were identified. Using single-particle cryo-EM and helical reconstruction, we obtained a 2.9-Å resolution structure and confirmed that the sheath is composed of Cis2 (Fig. 1, C and D). Except for the flexible region between domains 2 and 3 (residues 209 to 291), all residues could be built into the final atomic model.

By comparing the *Hb* Cis2 structure against eCIS homologs from *Serratia entomophila* (*21*), *Photorhabdus asymbiotica* (*22*), *Algoriphagus machipongonensis* (*24*), the cytoplasmic CIS homolog from *Streptomyces coelicolor* (*28*), and T6SS homologs from *Candidatus* Amoebophilus asiaticus (*29*) and *Aureispira* sp. CCB-QB1 (*30*) (both generated by AlphaFold), we identified that the largest structural variation is found in domain 3 (fig. S1, C to H). In contrast, domains 1 and 2, which face the inside of the assembled sheath and facilitate the contractile mechanism, show a low root mean square deviation (RMSD) of around 1.0 to 1.7 Å. The Cis2 homolog from *S. coelicolor* showed the highest structural similarity for both the entire structure (4.1-Å RMSD) and only domains 1 and 2 (0.8 Å-RMSD). This was supported by a Dali search (*31*), retrieving Cis2 [Protein Data Bank (PDB): 8BKY] from *S. coelicolor* as the top hit (*Z* score: 35.7).

### *H. borinquense* exhibits antagonism toward *Haloferax volcanii*

To obtain functional insights into the *Hb* CIS, we first generated antibodies against the putative inner tube (Cis1) and sheath (Cis2). Using Western blotting, we then monitored changes in expression over the course of 72 hours. Sheath expression remained relatively stable during the exponential phase, while protein levels for the inner tube started dropping rapidly after the mid-exponential phase (fig. S1, I and J). A similar change in abundance of the inner tube was previously observed for the *Vibrio cholerae* T6SS (*32*).

Having identified conditions for CIS expression, we then set out to probe if *Hb* exerted an antagonistic effect on other organisms using a killing assay. To perform killing assays under conditions that are compatible with both *Hb* and the prey, we chose to use the haloarchaeon *Haloferax volcanii* (hereafter *H. volcanii* or *Hv*). *Hv* does not harbor a CIS gene cluster and is genetically tractable, which made it an excellent strain for analyzing cell-cell interactions in liquid and on solid media.

To perform killing assays, both strains were first grown to the early exponential phase and then mixed together. We chose a ratio of 1:5 (*Hv*:*Hb*) to increase the number of *Hb* cells in direct contact with each *Hv* cell and to promote any potential effects that the CIS would have in cell-cell interactions. During liquid killing assays, the mixture was incubated shaking for 24 hours, and for solid killing assays, the mixture was spotted onto a solid medium and incubated for 24 hours. To quantify these killing assays, cells from liquid and solid killing assays were serially diluted and spotted onto a solid medium, which specifically selected for *Hv*. The results of the liquid killing assay showed only a slight decrease in *Hv* growth compared to the control. The solid killing assay, on the other hand, revealed a substantial drop in *Hv* growth by more than 1000-fold (Fig. 1E). This seemed to indicate that the transient interactions between *Hv* and *Hb* in the liquid killing assay were not as effective in leading to antagonism, as compared to the continuous interaction between the two strains during the solid killing assay.

### Antagonism is dependent on cell-cell contact and functional CISs

To better understand this antagonistic behavior, we studied cell-cell interactions on a single-cell level. We applied time-lapse imaging to a 1:2 mixture of *Hv* cells expressing cytoplasmic mScarlet (hereafter *Hv*-mScarlet) and wild-type *Hb* cells, growing at 45°C for 960 min. During cell fate quantification, mScarlet was initially used to identify *Hv-*mScarlet cells at the start of a time series. However, the *Hv* mScarlet signal is lost after the first few frames during imaging. We postulate that this occurs due to the presence of *Hb*.

**Fig. 2.**
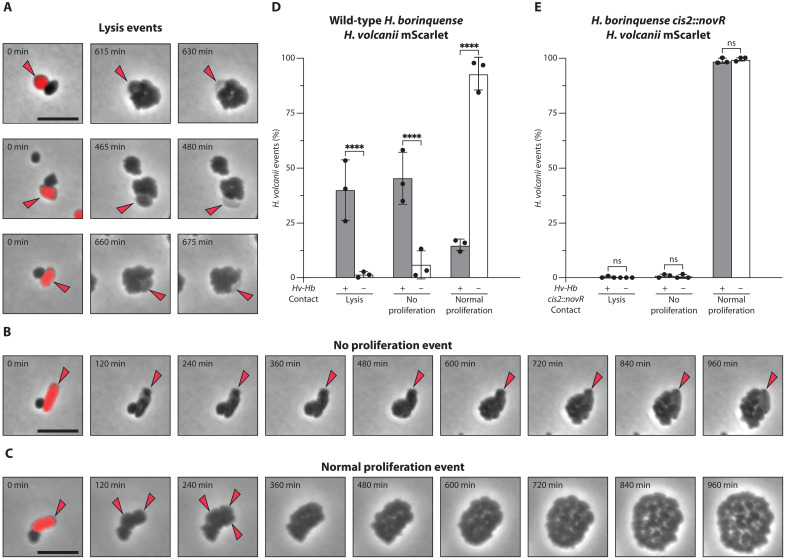
Antagonism is contact-dependent and CIS-dependent. (**A** to **C**) Time-lapse imaging (15-min frame rate, 960 min, 45°C) of a coincubation of *H. borinquense* with *H. volcanii* mScarlet. Shown are selected frames as overlays of phase contrast with red fluorescence (mScarlet). Because of the substantial drop in mScarlet signal after the first time point, arrowheads are used to follow the fate of *H. volcanii* mScarlet cells. Three distinct classes of *H. volcanii* cell fates were observed upon cell-cell interactions: panel (A) shows three examples of cell lysis events, characterized by the loss of *H. volcanii* cellular integrity. (B) A no proliferation event, in which *H. borinquense* proliferates, while *H. volcanii* mScarlet proliferation is impaired. (C) A normal proliferation event of both strains. The growth of *Hv* was only followed with arrowheads in the first three images. Note that, for (C), the experiment was done with the sheath mutant *H. borinquense* c*is2*::*novR*. Scale bars, 5 μm. (**D**) Quantification of cell fates exhibited by *H. volcanii* mScarlet with and without contact to wild-type *H. borinquense*. Contact events saw a significant decrease in the number of normal proliferation events with a significant increase in both cell lysis and no proliferation events (****P < 0.0001; *N*^contact^ = 243; *N*^no contact^ = 332; mean with SD, two-way ANOVA). (**E**) Quantification of cell fates exhibited by *H. volcanii* mScarlet with and without contact to *H. borinquense cis2*::*novR*. Contact between the two strains did not affect the number of *H. volcanii* mScarlet cells with normal proliferation events and did not lead to a significant increase in the number of cell lysis nor no proliferation events (ns > 0.9999; *N*^contact^ = 343; *N*^no contact^ = 320; mean with SD, two-way ANOVA).

During time-lapse imaging, we identified three distinct *Hv*-mScarlet cell fates: “cell lysis,” “no proliferation,” and “normal proliferation” (Fig. 2, A to C, and movies S1 and S2). We first quantified the fates of *Hv*-mScarlet cells that were in contact with *Hb*. This revealed a substantial number of interactions, in which *Hv*-mScarlet cells lysed (41.6%, *n* = 332) or no longer proliferated (43.7%, *n* = 332), with a total 85.3% of the interactions being antagonistic (Fig. 2D). In contrast, *Hv*-mScarlet cells that were not in contact with *Hb* during imaging primarily underwent normal cell proliferation (95.1%, *n* = 243), with only 4.9% of the cells lysing or not proliferating. This suggests that the observed antagonistic effect on *Hv*-mScarlet relies on direct cell-cell contact with *Hb*, which is consistent with findings from the solid killing assay (Fig. 1E).

To determine the role of the CIS in this antagonistic interaction, we set out to disrupt the CIS gene cluster and test the resulting mutant in time-lapse imaging. While there are no established genetic tools for manipulating *Hb*, closely related haloarchaea are known to perform homologous recombination (*33*). Therefore, by transforming a plasmid with a novobiocin resistance gene flanked by sheath gene fragments, we were able to trigger homologous recombination and disrupt the sheath gene in *Hb* (hereafter *Hb-cis2*::*novR*). The absence of proper CIS assembly in the generated mutant was verified via sequencing, sheath purifications in which the typical sheath structures were no longer observable, and Western blots that showed that the full-length sheath protein was no longer expressed (fig. S2). In addition, Western blotting also found that inner tube (Cis1) expression was impaired by the homologous recombination event, likely due to the gene’s location directly downstream of *cis2*. Together, these findings indicate that the mutant was no longer able to assemble functional CISs. We then tested the interaction between *Hb*-*cis2*::*novR* and *Hv*-mScarlet using time-lapse imaging. Notably, *Hv*-mScarlet cell lysis and no proliferation events were almost entirely absent (1.2% for *Hv*-mScarlet cells in contact with *Hb*-c*is2*::*novR*, *n* = 343; 0.6% for *Hv*-mScarlet cells without contact with *Hb*-c*is2*::*novR*, *n* = 320) (Fig. 2E). These findings suggest that both direct cell-cell contact and CIS assembly are required for the antagonistic activity exerted by *Hb* on *Hv-*mScarlet.

### The archaeal CIS binds to the cytoplasmic membrane in a T6SS-like manner

**Fig. 3.**
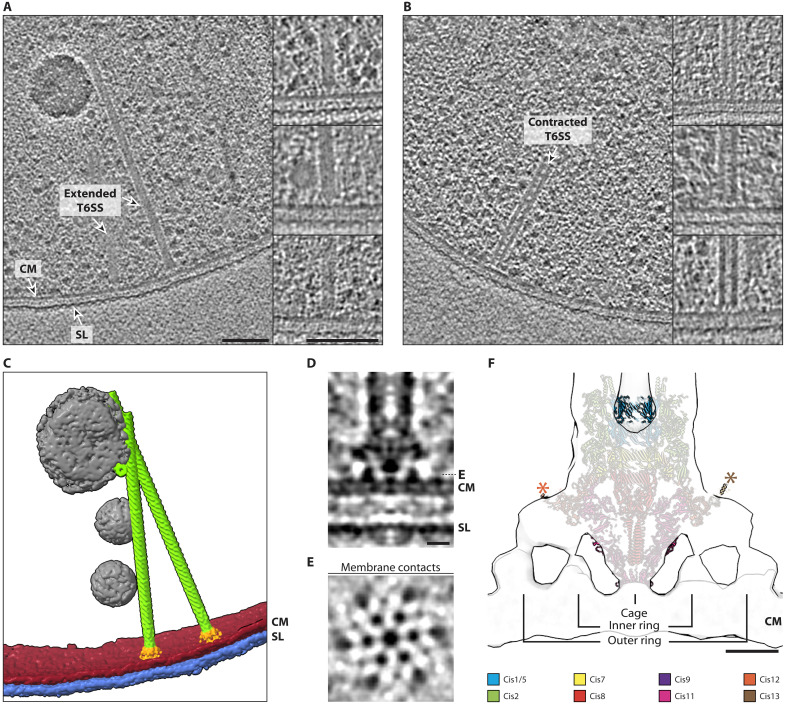
CIS binds to archaeal membrane in a T6SS-like manner. (**A** and **B**) Slices through tomograms collected on cryo-FIB–milled *H. borinquense* cells grown at 45°C and 2.45 M NaCl. Tomograms reveal extended (A) and contracted (B) T6SS-like CIS structures inside the cells. Additional examples of both extended and contracted T6SSs are also shown. Shown are 13.6-nm-thick slices through Tegunov deconvolution-filtered tomograms. CM, cytoplasmic membrane; SL, S-layer. Scale bars, 100 nm. (**C**) Segmentation of the cryo-tomogram depicted in (A), with the cytoplasmic membrane (CM) in red, S-layer (SL) in blue, putative storage granules in gray, and the T6SS baseplate and sheath-tube module in orange and green, respectively. (**D** and **E**) Subtomogram average of C6-symmetrized extended T6SS baseplate and membrane anchor using 57 particles that were extracted from filtered tomograms. Shown are 1.36-nm-thick longitudinal (D) and perpendicular slices (E). The perpendicular slice reveals contacts with the membrane, i.e., densities of six feet-like structures that are arranged around the central cage. CM, cytoplasmic membrane; SL, S-layer. Scale bar, 10 nm. (**F**) Shown is a longitudinal slice through a Gaussian-filtered subtomogram average isosurface (gray) from (D) and (E). The extended baseplate of the tCIS from *Anabaena* sp. PCC 7120 [PDB: 7B5H (*27*)] was fitted into the average. tCIS proteins Cis19 and Cis6 were removed because the *H. borinquense* CIS gene cluster lacks homologs of these proteins. Note the good fit of Cis11, which makes contact with the membrane. Cis12 and Cis13 (highlighted with asterisks) at the periphery of the baseplate may also be involved in membrane anchoring. Scale bar, 10 nm.

Time-lapse imaging highlighted the importance of the *Hb* CIS for contact-dependent antagonism. To understand how the CIS architecture and mode of action contribute to its function, a combination of cryo–focused ion beam (cryo-FIB) milling and cryo–electron tomography (cryo-ET) was applied to whole *Hb* cells. Within the resulting tomograms, CISs were observed in both extended (~16 nm in diameter) and contracted (~19 nm in diameter) states (fig. S3, A and B). Extended CIS structures, in which the baseplate was identifiable, were always perpendicular to the cytoplasmic membrane with a putative membrane anchoring structure (Fig. 3, A and C). Contracted sheaths were observed as either free-floating or perpendicular to the cytoplasmic membrane (Fig. 3B). On the basis of the T6SS-like subcellular localization of the CIS, hereafter, we will refer to the system as an archaeal T6SS.

The *Hb* cell envelope architecture is fundamentally different to that of Gram-negative bacteria with T6SSs, and it is composed of a cytoplasmic membrane that is associated with an ordered layer of glycosylated protein—called S-layer (*34*, *35*). We were therefore interested in how a T6SS evolved to make contact with this distinct type of cell envelope. Unfortunately, the high salt concentrations that are required to propagate *Hb* resulted in a poor signal-to-noise ratio. This made characterizing the structural modules of the T6SS particularly difficult. We set out to improve the contrast of the baseplate region by subtomogram averaging. Because of the structural heterogeneity of contracted T6SSs, possibly due to the presence of disassembly intermediates, we focused on generating an average of the extended state. Initial averages identified a sixfold rotational symmetry in the sheath-tube, baseplate, and membrane anchoring modules (fig. S3, C to E). We therefore applied C6 symmetry during the final rounds of subtomogram averaging (fig. S3F).

Resolving the contact between the membrane and the baseplate was still difficult; therefore, subtomogram averaging was performed using deconvolution-filtered data to improve the contrast. The resulting average (Fig. 3, D and E) showed the same features; however, it allowed better visualization of the baseplate and membrane anchor. Both averages did not permit individual subunits of the baseplate to be resolved, but six “feet-like” structures emanating from the periphery of the baseplate and a central “cage” at the base of the baseplate are clearly visible. Each individual “foot-like” structure makes contact with the archaeal cytoplasmic membrane in two locations, forming an outer and inner ring of membrane attachment points that center around the cage, which also contacts the cytoplasmic membrane at the center of the T6SS.

To identify the proteins likely involved in these membrane contacts, the high-resolution structure of the prefiring baseplate from *Anabaena* sp. PCC 7120 (hereafter tCIS) (*27*) was docked into the subtomogram average from deconvolved data (Fig. 3F). The tCIS was chosen due to the presence of all the structural proteins found in the *Hb* CIS gene cluster and because it also anchors itself to membranes in situ. In addition, it is one of the most closely related CIS gene clusters that have been studied. The structures showed a high overall similarity at the baseplate (Cis1/2/5/7/8/9/12/13) and the cage (Cis11). The fitting permitted us to postulate that Cis12 and Cis13 are likely involved in membrane attachment as both fit within the peripheral densities of the baseplate. The additional contact made at the base of the baseplate also correlates well with the cage formed by Cis11. Because of the presence of two *cis11* genes in the *Hb* gene cluster, the cage is likely formed by either one or both of these gene products. From the subtomogram average, it was impossible to determine how the CIS attaches to the membrane. Cis11, 12, and 13 may interact directly with the membrane or via a membrane-embedded protein that was not resolved during averaging.

This mechanism of attaching to membranes via six feet-like structures is also observed for the T6SS subtype iv found in *Candidatus* Amoebophilus asiaticus (*29*); however, the overall architecture of membrane anchoring looks drastically different.

### *H. borinquense* encodes T6SS toxin-immunity pairs

Toxin translocation is a hallmark of a functional T6SS (*36*, *37*). However, the identification of toxins can be a challenging, especially if these toxins are not associated with the T6SS gene cluster. By using a two-pronged approach, first, we attempted to determine if proteins within the interspacing region of the T6SS gene cluster are toxic. Second, by analyzing the secretome of *Hb*, we sought to identify further potential T6SS toxins found in the *Hb* genome.

**Fig. 4.**
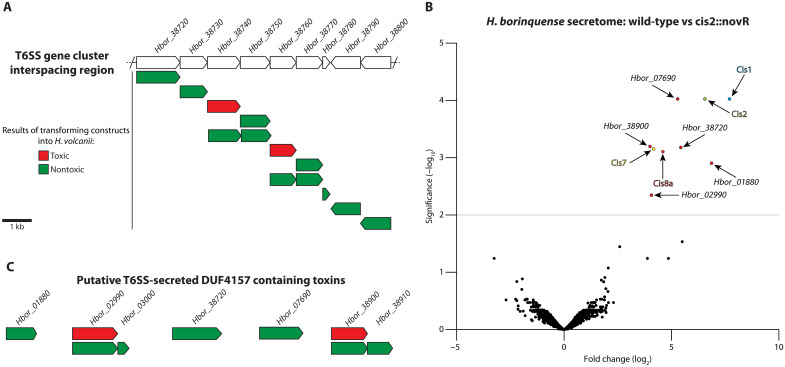
*H. borinquense* secretes toxic proteins in a T6SS-dependent manner. (**A**) Schematic of the interspacing region in the middle of the *H. borinquense* T6SS gene cluster. Each of these genes was expressed in *H. volcanii*. Of these genes, two were identified to be toxic (*Hbor_38740* and *Hbor_38760*, depicted in red). The expression of these two toxic genes in tandem with their downstream “partner” was able to prevent toxicity. (**B**) Volcano plot generated by comparing the secretomes of wild-type *H. borinquense* against the *H. borinquense* sheath mutant *cis2*::*novR*. Nine genes were identified to be significantly more abundant in the wild-type secretome. Four of these genes (*Hbor_01880*, *Hbor_02990*, *Hbor_07690*, *Hbor_38720*, and *Hbor_38900*) have a DUF4157 domain. (**C**) Each of the DUF4157 domain containing proteins, identified in the secretome analysis, was expressed in *H. volcanii*. Two of these genes (*Hbor_02990* and *Hbor_38900*, depicted in red) were toxic when expressed. The expression of these genes in tandem with their downstream partner (depicted in green) was able to prevent self-intoxication.

The interspacing region contains nine genes and is found in the middle of the *Hb* T6SS gene cluster (Figs. 1A and 4A). Bioinformatics analysis of these genes did not identify any obvious toxin candidates. *Hbor_38720* and *Hbor_38740* do, however, have a predicted DUF4157 domain that has been identified within some other CIS-related cargoes (*24*, *38*). By using a plasmid generated in this study (pWL102et), we attempted to express each of the interspacing region genes in *Hv*. Transformation of the generated plasmids into *Hv* was successful for all genes, except for *Hbor_38740* and *Hbor_38760* (Fig. 4A). Repeated attempts to transform plasmids with these two genes resulted in either the absence of any transformants or excision of the respective gene in the plasmids of successful transformants, indicating that the gene products may be toxic in *Hv*. To prevent self-intoxication and toxicity from sister cells, T6SS associated toxins are commonly associated with immunity genes (*36*). To test if this was true for the identified toxins, we designed constructs to express *Hbor_38740* and *Hbor_38760* in tandem with their downstream partners *Hbor_38750* and *Hbor_38770*, respectively. These constructs were successfully transformed, and the expression of the putative immunity gene product was detectable (Fig. 4A and fig. S4A).

In an effort to identify additional toxins, we also used a system-level approach by analyzing the differences in the secretomes of wild-type *Hb* and *Hb-cis2*::*novR*, which no longer assembles functional T6SSs. Comparison of the relative protein abundances found nine gene products that were secreted at significantly higher levels in wild-type *Hb* (Fig. 4B). *Hbor_38740/Hbor_38760* were not among these gene products, potentially indicating that they are only produced/secreted under specific conditions. Of the nine proteins that were identified, four are key T6SS structural components (Cis1/2/7/8a), while the remaining five proteins (*Hbor_01880*, *Hbor_02990*, *Hbor_07690*, *Hbor_38720*, and *Hbor_38900*) all contain a DUF4157 domain. Most notable were *Hbor_38720* and *Hbor_38900*, which are located within the interspacing region or directly upstream of the *Hb* T6SS gene cluster, respectively. The identification of *Hbor_38720* was particularly interesting as it was not found to be toxic in our first approach (Fig. 4A), which could indicate that it may not act as a toxin against *Hv*.

By using plasmid pWL102et, we attempted to express each of the identified DUF4157 domain-containing proteins in *Hv.* Similar to the previously identified toxins (Fig. 4A), transformation of *Hbor_02990* and *Hbor_38900* into *Hv* was unsuccessful, resulting in either the absence of any transformants or in transformants that contained a plasmid where the respective gene was excised (Fig. 4C). This led us to postulate that these two genes are also potential toxins against *Hv*. Furthermore, both *Hbor_02990* and *Hbor_38900* were closely associated with a downstream “immunity” partner that seemed to prevent toxicity when expressed in tandem with their respective toxin. It should be noted that, although *Hbor_01880* and *Hbor_38720* were not found to be toxic in *Hv*, both genes are closely associated with a downstream gene, potentially indicating that they may be toxic against another target organism.

Together, we identified six putative CIS-associated toxin-immunity pairs, while the functional role of four of these was verified in *Hv*.

### Other haloarchaeal species express a CIS and exhibit contact-dependent antagonism

**Fig. 5.**
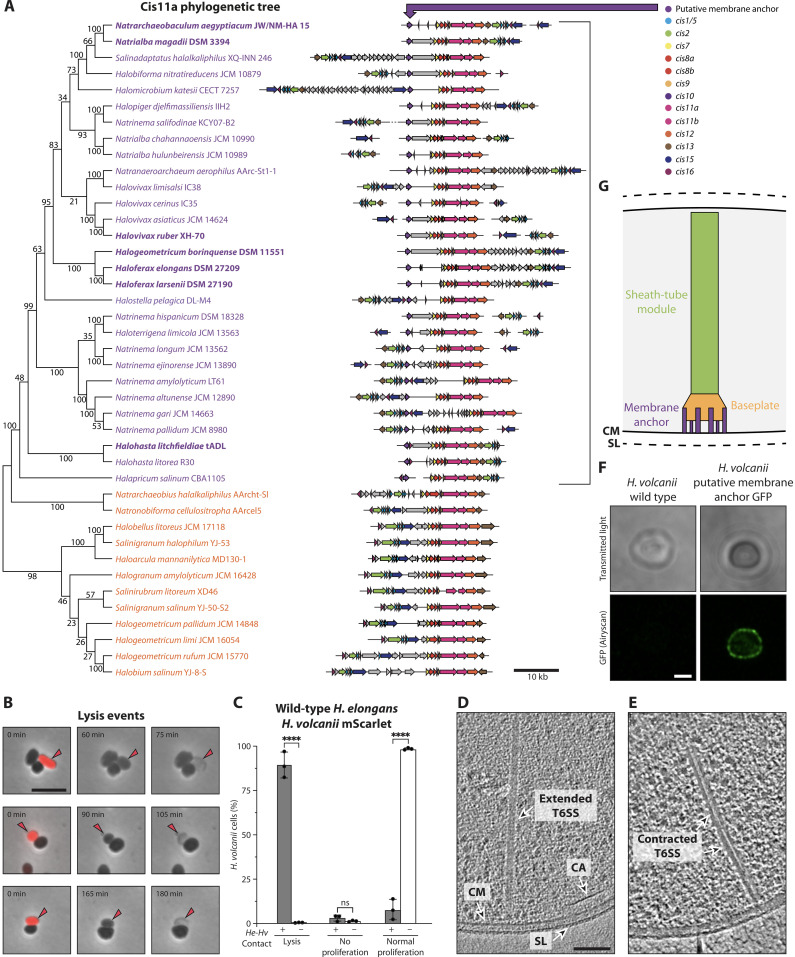
Haloarchaeal CIS gene clusters are conserved and a subset functions via a T6SS mode of action. (**A**) Phylogenetic tree (with bootstraps) of haloarchaeal Cis11a homologs paired with schematics of their CIS gene clusters, revealing two stable clades (purple/orange) and similar synteny. Clusters from the purple clade encode an additional conserved putative membrane protein. Bold font indicates species for which CIS expression was verified. (**B**) Time-lapse imaging (15-min frame rate, 960 min, 45°C) of a coincubation of *H. elongans* and *H. volcanii* mScarlet. Shown are selected frames as overlays of phase contrast with red fluorescence (mScarlet). Because of the substantial drop in fluorescence after the first time point, arrows are used to follow the fate of *H. volcanii* mScarlet. Shown are three examples of cell lysis events. Scale bar, 5 μm. (**C**) Quantification of cell fates exhibited by *H. volcanii* mScarlet with and without contact to *H. elongans*. Contact events saw a significant increase in cell lysis events (ns > 0.9999; ****P < 0.0001; *N*^contact^ = 321; *N*^no contact^ = 1235; mean with SD, two-way ANOVA). (**D** and **E**) Slices through tomograms of *H. elongans* cells grown at 45°C and 2.45 M NaCl. (D) An extended T6SS-like CIS. (E) A contracted T6SS. Shown are 13.6-nm slices through filtered tomograms. CM, cytoplasmic membrane; SL, S-layer; CA, chemotaxis array. Scale bar, 100 nm. (**F**) Representative images of *H. volcanii* wild-type and *H. volcanii* expressing the putative GFP-tagged membrane anchor *Hbor_38620*. Top row (minimum intensity *z* projections of transmitted light) and bottom row (confocal GFP slice) indicate that *Hbor_38620* localizes to the cell envelope. Scale bar, 1 μm. (**G**) Schematic of the haloarchaeal T6SS. Cryo-ET imaging, functional assays, Cis11a phylogeny, and gene cluster analyses indicate that representatives of the purple clade may all have a T6SS mode of action, mediated via *Hbor_38620* homologs.

The characterization of an archaeal T6SS in *Hb* revealed a molecular mechanism by which archaea can interact with their environment. To determine if the observed function and mode of action are conserved, we explored the abundance of CIS gene clusters in archaea. We focused on identifying CISs in the class *Halobacteria* (halophilic archaea), for which we have established functional assays to characterize archaea-archaea interactions. From a total of 350 analyzed genomes, 41 strains (12%) harbor a gene cluster that contains the structural genes required for assembling a functional CIS (*cis*1-16) (Fig. 5A). These gene clusters were detected in three of four phylogenetic orders (fig. S5A), with all representatives having a rather well-conserved gene content and synteny (Fig. 5A). In seven of eight complete genomes, the CIS gene cluster is located on the main replicon. The phylogenetic trees generated from 16*S* ribosomal DNA (rDNA) and Cis11a sequences did not match (Fig. 5A and fig. S5A), indicating horizontal gene transfer of CIS gene clusters among halophilic archaea. To better understand the origin of the haloarchaeal CIS gene clusters, we generated a phylogenetic tree that contained Cis11 representatives from 1520 bioinformatically identified bacterial CIS gene clusters [from (*14*)]. This tree reveals that the closest relatives of the haloarchaeal Cis11 homologs are representatives from the Gram-positive Bacillota (Firmicutes) (fig. S6), supporting our insights from Cis2 structure comparisons (fig. S1F) and indicating a possible horizontal gene transfer of the gene cluster.

To test if other haloarchaea express a CIS, we performed crude CIS purifications of 16 CIS-encoding strains. Using negative stain transmission electron microscopy (TEM), we could verify that sheath-like structures were expressed in six additional strains (bold species names in Fig. 5A and fig. S5A). Among the identified CIS-positive strains was *Haloferax elongans* (hereafter *H. elongans* or *He*), which showed the highest abundance of observable sheath structures. Because *He* grows under the same conditions as *Hv*, we proceeded to study their interaction by time-lapse imaging. Similar to our observations of coincubating *Hb* with *Hv-*mScarlet (Fig. 2), a coincubation of *He* with *Hv-*mScarlet (2:1 ratio) revealed cell lysis, no proliferation, and normal proliferation cell fates (Fig. 5, B and C). Contact with *He* led to a significantly higher percentage of *Hv*-mScarlet cell lysis events (89.1%, *n* = 321 for *He* versus 41.6%, *n* = 332 for *Hb*), with a total of 92.2% of interactions being antagonistic (Fig. 5C). *Hv-*mScarlet cells growing without coming in contact with *He* had an insignificant number of antagonistic-like cell fates (1.3%, *n* = 1235). It should be noted that the conditions during imaging were not optimal for *He* and led to occasional cell lysis of *He* even in the absence of *Hv*-mScarlet (fig. S7).

The contact-dependent antagonism indicated the possibility of a T6SS mode of action in *He*. We verified this theory by imaging *He* cells by cryo-ET. The resulting tomograms showed *He* T6SSs in both the extended and contracted state. For structures in the extended state, the T6SSs were always attached to the cytoplasmic membrane in a visually similar manner as observed for the *Hb* T6SS (Fig. 5D). T6SSs in the contracted state were less common and found free-floating or perpendicular to the cell envelope (Fig. 5E). Together, the time-lapse imaging and in situ structural data indicate that the *He* CIS is an additional example of an archaeal T6SS that likely mediates contact-dependent antagonism.

### The T6SS mode of action correlates with a conserved putative membrane anchor

The identification of two CIS gene clusters with a T6SS mode of action indicates an evolutionary drive toward anchoring these systems to the archaeal cell envelope. Therefore, we further analyzed the haloarchaeal CIS gene clusters for potential membrane-anchoring proteins. The Cis11a phylogenetic tree revealed two distinct, stable groups of representatives (labeled purple and orange in Fig. 5A). The gene clusters corresponding to the purple group, with 29 representatives (including Cis11a from *Hb* and *He*), were always associated with an uncharacterized conserved gene. In *Hb* and *He*, this gene (*Hbor_38620* and *SAMN04488691_10831*, respectively) is found upstream of the core CIS gene cluster. Bioinformatics characterization of *Hbor_38620* identified six predicted transmembrane helices and a ~150-residue-long cytoplasmic domain (fig. S5, B and C), indicating a putative role in anchoring the T6SS to the cytoplasmic membrane. The absence of *Hbor_38620* homologs in the second group of CIS gene clusters (orange in Fig. 5A) may suggest a different mode of action for these systems.

To test if *Hbor_38620* functions as a membrane protein, we green fluorescent protein (GFP)–tagged and expressed *Hbor_38620* in *Hv*. Using confocal microscopy, we observed that *Hbor_38620* localized to the cell membrane (Fig. 5F). The localization of *Hbor_38620* at the cell membrane coupled with the association of *Hbor_38620* homologs to CIS gene clusters with a T6SS mode of action could hint at its potential role in anchoring the archaeal T6SS to the membrane (Fig. 5G).

## DISCUSSION

Archaea are known to interact with other microorganisms; however, antagonistic interactions and their underlying mechanisms are largely unexplored. In this work, we studied an archaeal CIS in *H. borinquense*. Characterization of both its structure and function gives insights into the evolution of T6SSs and furthers our understanding of the roles that archaea have in their environment.

Our first major insight was that the CIS gene cluster in *Hb* has a T6SS mode of action, i.e., it functions while bound to the cytoplasmic membrane. However, its architecture is different to the previously described T6SSs. In the canonical T6SSs subtypes i to iii (*39*–*41*) and the recently reported T6SS subtype iv found in *Aureispira* sp. CCB-QB1 (*30*), a large trans-envelope complex spans between the inner and outer membrane. Likely as an adaptation to the archaeal cell envelope, which features an S-layer instead of a cell wall and outer membrane, the T6SS in *Hb* does not seem to have a complex outside of the cytoplasmic membrane. The system therefore appears rather minimal, akin to the T6SS subtype iv in the Gram-negative bacteria *Candidatus* Amoebophilus asiaticus (*29*). Despite this overarching similarity, however, the interactions between the baseplate and membrane are different. Subtomogram averaging of the *Hb* T6SS revealed that the interactions between the T6SS and the membrane are mediated via the cage, which surrounds the spike, as well as by six feet-like densities emanating from the periphery of the baseplate (Fig. 3E). In the T6SS subtype iv of *Amoebophilus*, the feet-like structures emanate from the base of the baseplate rather than from the periphery as seen in *Hb*.

From the *Hb* T6SS subtomogram average, we also identified that the feet-like densities are likely not fully accounted for by the conserved baseplate components Cis11/12/13 (Fig. 3F). We speculate that the putative membrane anchor *Hbor_38620* may interact with components of the baseplate to facilitate T6SS localization at the cell envelope. *Hbor_38620* homologs are not present in CIS-negative haloarchaea. Furthermore, from the 41 haloarchaeal CIS gene clusters, we identified a distinct subset of 29 strains that all share this putative membrane protein, including the T6SS in *He*. The distinction between the two clades becomes further evident by the comparison of predicted Cis2 structures, with the highest Cis2 similarities being detected between representatives that encode a homolog of *Hbor_38620* (fig. S8). The presence of *Hbor_38620* homologs may be useful for predicting a T6SS mode of action in uncharacterized CIS gene clusters of haloarchaea.

Our second major finding was that *Hb* disrupts proliferation and induces cell lysis of *Hv* in a contact-dependent and T6SS-dependent manner. This contact-dependent antagonistic behavior was also verifiable in *He*. Antagonism has been observed in various archaeal species through the secretion of small peptide archaeocins into their surrounding environment (*9*, *11*). However, the targeted delivery of toxins from one archaeon to another has not been observed to date. Such a toxin delivery system has implications in the study of archaeal biofilms and other niches, in which archaea reside in close contact (*4*). The intragenus interspecific killing on surfaces could also represent a potential speciation barrier in this group of organisms.

During the analysis of the *Hb* T6SS gene cluster and secretome, we also identified six putative toxin-immunity pairs. Four of these pairs were associated with the T6SS gene cluster, while another two are found elsewhere in the *Hb* genome. By expressing each of these toxins in *Hv*, we were able to verify that four of these proteins are toxic toward *Hv* and we also showed that the expression of these toxins in tandem with their downstream immunity protein prevented toxicity. This is similar to toxin-immunity pairs found in canonical T6SSs (*20*, *42*). From tomography data and subtomogram averaging, we did not observe extra densities within the lumen of the inner tube. We therefore postulate that these toxins attach to the T6SS spike, as was previously shown (*43*, *44*).

In conclusion, the insights and methods used in this study provide a framework for studying distinct types of archaeal cell-cell interaction in the future. It will be exciting to explore the ecological advantage that haloarchaea may have acquired by expressing CIS gene clusters in complex microbial communities. One area, which was not explored in this study, is the presence of CIS gene clusters in methanogenic and anaerobic methanotrophic archaea (*11*–*13*). These distant relatives to haloarchaea also thrive in close interactions with other species. It will be exciting to see what advantage the CIS provide in these ecological niches.

## MATERIALS AND METHODS

### Strains and culturing conditions

The archaeal strains studied in this work and their growth conditions are found in table S2. The archaeal media YPC and Ca are based on the previously described *Hv*-YPC and *Hv*-Ca media, respectively (*45*). Growth media with a “DSM” number are from the German Collection of Microorganisms and Cell Cultures, and those with a “JCM” number are from the Japanese Collection of Microorganisms. Cultures were grown in 5-ml aeration tubes, and larger volumes were grown in flasks shaken at 200 rpm unless stated otherwise. Listed growth media were solidified, when necessary, with 1.5 to 2% agar. Archaeal strains used in functional assays and the generated *Hb-*c*is2*::*novR* strain were selected for with varying concentrations of the antibiotics lovastatin (LovR) and novobiocin (NovR). Additional information about the growth of each strain can be found in the preceding methods sections. The *Escherichia coli* strain Top10 was used to generate and propagate all constructs used in this study. Dam and Dcm methylase-deficient K12 *E. coli* was used to prepare unmethylated DNA required for archaeal transformations. Both bacterial strains were grown in LB media or agar substituted with ampicillin (100 μg ml^−1^).

### Plasmids and genetic manipulation of haloarchaea

All plasmids that were generated in this work are based on the backbones of plasmid pWL102 (DSM 5717) or pET15b (Novagen). The full list of generated plasmids and the oligonucleotides used in this work are summarized in tables S3 and S4, respectively. Transformations of plasmids into *Hv* and *Hb* were performed as previously described (*45*). In brief, cells were spheroplasted in a spheroplasting solution [1 M NaCl, 27 mM KCl, 50 mM tris-HCl (pH 8.5), and 15% sucrose] with 0.5 M EDTA. Subsequently, 30 μl of a DNA solution [10 μl of ~1 to 2 μg of unmethylated DNA, 15 μl of an unbuffered spheroplasting solution 1 M NaCl, 27 mM KCl, and 15% sucrose (pH 7.5), and 5 μl of 0.5 M EDTA (pH 8.0)] was mixed with the spheroplasted cells. To begin the transformation, equal volume polyethylene glycol, molecular weight 600 (PEG-600) (60% PEG-600 and 40% unbuffered spheroplasting solution) was gently added to the cells. Spheroplasting was stopped by adding a spheroplasting dilution solution [3.1 M NaCl, 113.2 mM MgCl_2_·6H_2_O, 108.9 mM MgSO_4_·7H_2_O, 72 mM KCl, 15.3 mM tris-HCl (pH 7.5), 15% sucrose, and 3 mM CaCl_2_]. Cells were then added to a regeneration solution [2.5 M NaCl, 88.6 mM MgCl_2_·6H_2_O, 85.2 mM MgSO_4_·7H_2_O, 56.3 mM KCl, 12 mM tris-HCl (pH 7.5), 1.25 g of yeast extract, 0.25 g of peptone, 0.25 g of casamino acids, 15% sucrose, and 3 mM CaCl_2_]. To recover, cells were left to incubate at 45°C for 2 hours without shaking and subsequently 4 hours with shaking. YPC solid media supplemented with lovastatin or novobiocin was used to select for successful transformations. Transformation of plasmids, with a pWL102 backbone, used selective media containing lovastatin (either 0.5 or 4 μg ml^−1^). Plasmids with a novobiocin resistance gene were selected for with novobiocin (0.3 μg ml^−1^). To verify that plasmids had been successfully transformed or that homologous recombination occurred, polymerase chain reaction (PCR) was used.

### Sheath preparation of haloarchaea for negative stain and mass spectrometry

To isolate sheath, archaeal strains were grown for 24 hours (*Hb* and *Hb-*c*is2*::*novR)* or 72 hours (CIS expression tests) in 25 ml of their respective media (table S2). Cells were then pelleted (7000*g*, 10 min) and resuspended in 3 to 5 ml of a lysis buffer [150 mM NaCl, 50 mM tris-HCl, 0.5x CellLytic B (Sigma-Aldrich), 1% Triton X-100, and DNAse I (50 μg ml^−1^) (pH 7.4)] and incubated for 30 to 60 min at 37°C. Cell debris was removed via centrifugation at 15,000*g* for 15 min at 4°C. To isolate the sheath structures, the supernatant was ultracentrifuged at 150,000*g* for 1 hour at 4°C. The resulting pellet was resuspended in 25 to 50 μl of a resuspension buffer [150 mM NaCl and 50 mM Tris (pH 7.4)] and immediately used for EM. To identify proteins in the sheath preparation, samples were sent for mass spectrometry analysis at the Functional Genomics Center Zürich. Samples were prepared for mass spectrometry by precipitating proteins in a 30-μl sample using 10% trichloroacetic acid (TCA). The resulting precipitant was pelleted and washed twice with cold acetone. After drying the pellet, it was dissolved in 45 μl of a buffer [10 mM Tris and 2 mM CaCl_2_ (pH 8.2)] and 5 μl trypsin (100 ng/μl in 10 mM HCl) to digest the protein and then microwaved for 30 min at 60°C. Samples were dried and dissolved in 20 μl of 0.1% formic acid, diluted 1:10, and transferred to autosampler vials. Two microliters was injected for liquid chromatography with tandem mass spectrometry analysis. Database searches were performed by using the Mascot (swissprot, all species, and trembl_archaea) search program. For search results, stringent settings were applied in Scaffold (1% protein false discovery rate, a minimum of two peptides per protein, and 0.1% peptide false discovery rate). Mass spectrometry results were visualized with the software Scaffold (Proteome Software, v5.0.1).

### Negative stain EM

Three microliters of the sheath preparation sample was incubated for 1 min on glow-discharged carbon-coated Formvar grids (Electron Microscopy Sciences). To stain the sample, 3 μl of 1% phosphotungstic acid was added three times to the grid and left to incubate for 0, 15, and 30 s. To image the grids, an 80-kV Thermo Fisher Scientific Morgagni transmission electron microscope was used.

### Single-particle cryo-EM of the contracted *H. borinquense* sheath

Samples for single-particle cryo-EM analysis were prepared from a crude sheath preparation of *Hb* in the mid-exponential phase. The purified samples were directly used for vitrification with a Vitrobot Mark IV (Thermo Fisher Scientific) at 8°C and 100% humidity. A 4-μl sample was applied onto Au R2/2 200 mesh (Quantifoil) grids, and the excess liquid was then blotted away twice (2.5 s) after waiting for 15 s. The sample was then immediately plunged into liquid ethane/propane (37% v/v ethane). The vitrified grids were used for single-particle data collection.

Data collection was performed on a Titan Krios (Thermo Fisher Scientific) transmission electron microscope equipped with a K3 direct electron detector (Gatan) and Quantum LS GIF (Gatan, 20-eV slit). Data collection was performed using SerialEM (*46*). The potential contracted sheath particles were manually picked in low magnification and were used as a reference to align and collect data at high magnification (nominal magnification, ×81,000). Data were collected as a movie stack with 50 frames at super-resolution mode (an effective pixel size of 0.55 Å). The exposure time was 2.5 s, with an accumulated dose of ~65 e^−^/Å^2^. The movie frames of each collected stack were aligned and summed up into one micrograph with dose weighting at a binning factor of 2 using MotionCorr2 (pixel size of 1.10 Å) (*47*). The Contrast Transfer Function parameters were then estimated using Gctf (*48*). A total of 638 micrographs, with a defocus value ranging from −1 to −3.3 μm, were used during image processing.

The image processing was performed similar to previous reports (*24*, *27*). Briefly, the contracted sheaths were manually picked using Relion3.0 (*49*) with start-end coordinate pairs. The filaments were segmented with the inter-box distance of 18.3 Å. Bad particles were removed through one round of two-dimensional (2D) classification at a binning factor of 4, and particles in good 2D classes were used for 3D autorefinement applying sixfold symmetry but not helical symmetry. The initial helical parameters were then interpreted in real space using the relion_helix_toolbox and were further optimized in the following helical reconstruction (*50*). A total of 127,891 particles were used to reconstruct the contracted *Hb* sheath structure at a resolution of 3.4 Å, which was estimated based on the gold-standard Fourier shell correlation = 0.143 criteria (*51*). One additional round of CTF parameter optimization was performed, and the resolution was improved to 2.9 Å, assuming sixfold and helical symmetry (twist = 33.24°, rise = 17.09 Å).

### Atomic model building

The quality of the final reconstructed map was shown to be sufficient for de novo modeling. The atomic model of the contracted sheath was manually built using COOT (*52*). The model was iteratively refined using RosettaCM (*53*) and phenix.real_space_refine (*54*). The final atomic model was validated using phenix.molprobity (table S5). Structural illustrations and superpositions were performed using Chimera and ChimeraX (*55*, *56*). The Dali server was used to identify proteins that share structural similarities with *H. borinquense* Cis2 (*31*).

### Growth curve measurements

Late exponential phase cultures of *Hb* and *Hb-*c*is2*::*novR* were used to start fresh 25-ml YPC cultures at an optical density at 600 nm (OD_600nm_) of 0.01. Cultures were grown at 45°C, over 72 hours. Every 6 hours, a sample was taken from the culture to measure the OD_600nm_. To prepare samples for Western blotting, cells were pelleted to have 50 μl of an OD_600nm_ of 1.0. The pellet was resuspended in 1x Laemmli sample buffer (Bio-Rad), denatured for 10 min at 95°C, and stored at 4°C for Western blotting. Growth curve measurements were plotted using GraphPad Prism (v9.5.1).

### SDS-PAGE and Western blotting

A standard procedure was followed for running SDS–polyacrylamide gel electrophoresis (PAGE) and Western blots. In brief, samples were prepared to be at an OD_600nm_ of 1.0 in 50 μl of 1x Laemmli buffer (Bio-Rad). Samples were denatured for 10 min at 95°C, and then 10 to 30 μl was loaded onto a precast 4 to 15% protein gel (Bio-Rad). Electrophoresis was performed by applying a constant voltage of 200 V in a SDS running buffer (Tris/glycine/SDS, Bio-Rad) for ~1 hour. Samples were then transferred to a nitrocellulose membrane at 100 V for 1 hour at 4°C. The membrane was blocked with 5% milk TBST [10 mM tris-HCl (pH 8.0), 150 mM NaCl, and 0.05% Tween 20] at room temperature for 1 hour. For His-tagged samples, membranes were incubated with monoclonal primary anti-6xHis antibody (Sigma-Aldrich) in 1% milk TBST at 1:3000. To identify the expression of the *Hb* sheath (anti-Cis2) or inner tube (anti-Cis1), polyclonal antibodies were generated against *Hbor_38820* and *Hbor_38830*, respectively (GenScript). These primary antibodies were incubated in 1% milk TBST at 1:400. After incubation with the primary antibody for 2 hours at room temperature, the membranes were washed three times with TBST for 10 min. The secondary antibodies, anti-mouse for anti-6xHis (Merck) and anti-rabbit for anti-Cis1/2 (Invitrogen), both conjugated with horseradish peroxidase, were incubated with the membrane in 1% milk TBST at 1:10,000. After incubating at room temperature for 1 hour, the membranes were washed three times with TBST for 10 min. Membranes were imaged (ChemiDoc, Bio-Rad) after applying a chemiluminescent substrate (ECL, Bio-Rad).

### Solid-based and liquid-based killing assay

Late exponential phase cultures were used to start 25-ml YPC media cultures of *Hb* pWL102et and *Hv* pWL102et NovR at an OD_600nm_ of 0.01 with lovastatin (0.5 μg ml^−1^). Cultures were grown at 45°C for 24 hours. After 24 hours, *Hb* and *Hv* OD_600nm_ was measured and the strains were concentrated to 0.5 and 0.1, respectively. Five hundred microliters of *Hb* culture was then mixed with 500 μl of *Hv*. The mixtures were incubated for another 15 min before spotting 100 μl of each mixture onto YPC plates with lovastatin (0.5 μg ml^−1^). The remaining 900-μl mixtures were left at 45°C shaking for 24 hours (liquid killing assay). After the spots dried, the plates were also incubated at 45°C for 24 hours (solid killing assay). Each of the liquid killing assay mixtures was serially diluted (10^−2^, 10^−3^, 10^−4^, 10^−5^, and 10^−6^), and 25 μl of each dilution was spotted on YPC plates supplemented with novobiocin (0.3 μg ml^−1^). The spots of the solid killing assay were resuspended with 1 ml of YPC media, serially diluted, and spotted on YPC plates supplemented with novobiocin (0.3 μg ml^−1^) in the same manner as the liquid killing assay. After the spots dried, the plates were incubated at 45°C for about a week before taking colorimetric images (ChemiDoc, Bio-Rad).

### Time-lapse imaging functional assays

Late exponential phase cultures were used to start 25-ml Ca media cultures of *Hb* and *Hv-*mScarlet at an OD_600nm_ of 0.01. Cultures were grown at 45°C for 24 hours. After 24 hours, *Hb* and *Hv* were concentrated to an OD_600nm_ of 1.0 and 0.5, respectively. Each sample was left to equilibrate at 45°C for 15 min before mixing the two strains together for another 15 min. A 7.5-μl mixture was spotted onto an agarose pad [2.3 M NaCl, 88.3 mM MgCl_2_·6H_2_O, 85.3 mM MgSO_4_·7H_2_O, 56.5 mM KCl, 12 mM tris-HCl (pH 7.5), 3 mM CaCl_2_, and 1% agarose] and left to dry at room temperature. After 30 min, the agarose pad was placed in a glass-bottom imaging dish (μ-Dish, 35 mm high, Ibidi) and incubated at 45°C for 30 min. To prevent water evaporation and salt crystal formation while imaging, the imaging dish was closed with vacuum grease and parafilm. For imaging the sample, the Thunder Imager 3D cell culture microscope (Leica) i8 CO_2_ incubator (Pecon) was preheated to 45°C for at least 2.5 hours before imaging. To identify areas with good coverage of both *Hb* and *Hv-*mScarlet, a tile scan was performed using the Leica Application Suite X (LasX) software (v3.7.6.25997) using the HC PL APO 100x/1.40-0.70 oil objective (Leica). Images were collected using phase contrast to identify all cells and fluorescence (575-nm excitation) to identify mScarlet-expressing *Hv*-mScarlet. The *Hv-*mScarlet signal was quickly lost during imaging and therefore was only used in the first frame to determine which cells were *Hv*. Around 10 target areas were selected for time-lapse imaging. At each area, a *z* stack (2 μm, nine slices) was collected every 15 min over 16 hours. Fiji was used to make maximum intensity projections and to help in the quantification of the time-lapse imaging data (*57*). Imaging of the *Hb*-*cis2*::*novR* and *He* followed the same procedure. For the *Hb*-*cis2*::*novR*, however, the initial culture was supplemented with novobiocin (0.3 μg ml^−1^); however, novobiocin was not present during imaging. Graphs and statistical analyses were performed in GraphPad Prism. To quantify events, antagonism was characterized as an event in which *Hv* no longer proliferated or lysed without producing any daughter cells. Normal proliferation was described as an event where *Hv* continues to proliferate and generate at least one daughter cell.

### Vitrification of samples for cryo-ET

Samples for tomography data collection were prepared by growing *Hb* or *He* for 6 to 24 hours (~0.1 to 1 OD_600nm_) and then concentrating the cells to ~10 to 30 OD_600nm_ to create a lawn of cells on the generated grids. Four microliters of the concentrated cells was then spotted onto a glow discharged copper grid (R2/2, Quantifoil). By using a Vitrobot Mark IV (Thermo Fisher Scientific) plunge freezer with one blotting arm covered by Teflon, the excess liquid was removed by blotting the grid between 4 and 6 s from the back. Vitrification of the sample was achieved by using a mixture of ethane/propane (37% v/v ethane) cooled to liquid nitrogen temperature. Samples were subsequently stored in liquid nitrogen until sample thinning using cryo-FIB milling.

### Cryo-FIB milling

Cryo-FIB milling was performed with the automation methodology previously described (*58*). In brief, vitrified samples were clipped into modified autogrids (Thermo Fisher Scientific) and mounted onto a 40° pretilted cryo-FIB holder (Leica). The samples were then transferred to an ACE600 (Leica) using a VCT500 (Leica) and sputter coated with a ~4.5-nm layer of tungsten. Using the VCT500, the holder was subsequently mounted onto a cryo-stage (Leica) inside of a Crossbeam 550 FIB-SEM (Carl Zeiss Microscopy). To prepare the sample for the automated protocol, grids were coated with a layer of organoplatinum. Using the scanning electron microscope (SEM) (3 kV, 56 pA), milling targets were identified on the grids and then prepared for the automated milling procedure. During the automation, 12- to 20-μm lamellae were prepared using rough milling (100, 300, and 700 pA) and polishing currents (50 pA). We aimed for a final lamella thickness of ~200 to 250 nm. To save the milled sample for TEM imaging, grids were unloaded from the Crossbeam 550 using the VCT500 and stored in LN_2_.

### Cryo-ET data collection

Using cryo-ET, tomographic data were collected of the cryo-FIB–milled archaeal lawns. To record the data, we used a Titan Krios equipped with a Gatan K3 direct electron detector (Gatan) and GIF BioQuantum (Gatan, 20-eV slit). To automate tilt series collection, we used SerialEM to collect micrographs within a tilt range of 50° to −70° with 3° dose-symmetric increments. The total dose per tilt series was ~120 to 140 e^−^/Å^2^ with ~10 frames per tilt. The defocus used was ~8 μm with a pixel size of 3.4 Å.

### Tomogram reconstruction and subtomogram averaging

Collected tilt series were drift corrected using alignframes and reconstructed with the IMOD package (*59*). All shown tomograms are 4x binned and deconvolution filtered with tom_deconv (*60*). The segmentation of deconvolution filtered tomograms was done using a deep learning–based segmentation tool in Dragonfly (*61*). To perform subtomogram averaging, Dynamo was used for particle extraction, alignment, and averaging. To average the baseplate of the extended *Hb* CIS, an initial set of 68 particles were picked from cryo-FIB–milled *Hb* cells. Particles were extracted (88 by 88 by 88-pixel box size) from 4 by 4-binned tomograms (13.6 Å in pixel size) and aligned in six iterations. To remove poorly aligned particles, the particle dataset was cleaned based on cross-correlation values and aligned for another six rounds. The final average has C6 rotational symmetry and had a total of 57 unique extended CIS baseplate particles. The high-resolution atomic structure of the *Anabaena* PCC 7120 (PDB: 7B5H) extended CIS baseplate was fitted into the *Hb* subtomogram average, which was Gaussian filtered using ChimeraX (*56*). To generate an average of the extended and contracted sheath, 4522 extended and 3668 contracted sheath particles were extracted (40 by 40 by 40-pixel box size) from 4 by 4-binned cryo-FIB–milled tomograms (13.6 Å in pixel size). Particles were aligned over seven iterations with a final applied rotational symmetry of C6.

### Toxin-immunity gene identification and bioinformatics analysis

The genes identified in the interspacing region of the *Hb* CIS gene cluster (*Hbor_38720-38800*) were each amplified from the *Hb* cell lysate and ligated into the expression and C-terminal 6xHis-tagging plasmid pWL102et. Each generated plasmid was transformed into *Hv* using the transformation methodology previously described. To test if transformations were successful, colonies were tested for the presence of plasmid and insert using PCR. To determine what proteins might be secreted in a T6SS-dependent manner, late exponential phase cultures of *Hb* and *Hb-cis2*::*novR* were used to start 100-ml Ca cultures at an OD_600nm_ of 0.01. Cultures were grown at 45°C for 24 hours. These cultures were then spun down at 5000*g* for 10 min at 4°C, and the supernatant was subsequently filtered with a 0.2-μm filter on ice. To precipitate proteins, 25 ml of chilled TCA was added to the 100 ml of filtered culture for 1 hour at 4°C. The resulting mixture was spun down at 20,000*g* for 20 min at 4°C to pellet the proteins. Because of the high salt concentrations of the original media, the pellet was washed with 5 ml of chilled 20% TCA and then spun down at 20,000*g* for 20 min at 4°C. Afterward, 5 ml of acetone was used to wash the pellet and again spun down at 20,000*g* for 20 min at 4°C. The resulting pellet was air dried and sent for analysis at the Functional Genomics Center Zürich. For mass spectrometry analysis, samples were dissolved in a digestion buffer (10 mM Tris and 2 mM CaCl_2_), transferred to a 1.5-ml Eppendorf tube, and solubilized using high-intensity focused ultrasound. Protein concentration was estimated using the Lunatic UV/Vis absorbance spectrometer (Unchained Labs). Proteins were reduced and alkylated by adding tris(2-carboxyethyl)phosphine and chloroacetamide to a final concentration of 5 and 15 mM, respectively. The samples were incubated for 30 min at 30°C and 700 rpm and light protected. Digestion was done in a buffered trypsin solution at pH 8 (10 mM Tris and 2 mM CaCl_2_). Samples were enzymatically digested and dried. Peptides were acidified to perform a cleanup using home-made C18 StageTips. Peptides were loaded on the tip, washed, eluted, and dried to completeness. The digested samples were dissolved in aqueous 3% acetonitrile with 0.1% formic acid, and the peptide concentration was estimated with the Lunatic UV/Vis absorbance spectrometer (Unchained Labs). Peptides were separated on an M-class UPLC and analyzed on an Orbitrap mass spectrometer (Thermo Fisher Scientific) in data-independent acquisition (DIA). The acquired DIA spectra were processed with DIA-NN (version 1.8.2, PMID: 31768060) using a library-free approach with the *H. borinquense* proteome. The differential expression analysis verifies if the difference between normalized empirical protein abundances measured in two groups is significantly nonzero. A set of functions implemented in the R package prolfqua (*62*) were used to filter and normalize the data and to compute differential expression analysis. To further improve the power of the differential expression test, the protein variances are moderated, i.e., the individual protein variances are updated using a variance prior estimated from all the proteins in the experiment. GraphPad Prism (v9.5.1) was used to visualize the data.

In addition, Western blotting using anti-His antibodies was used to determine if there was expression from plasmids expressing the toxin in tandem with the immunity gene. Unsuccessfully, plasmid transformations were repeated three times and were also transformed into Top10 *E. coli*. To test if the identified toxins were accompanied by immunity genes, the toxin *Hbor_38740* and *Hbor_38760* were amplified together with their downstream partners *Hbor_38750* and *Hbor_38770*, respectively, using PCR. The amplified fragment was then transformed into *Hv*. PCR and Western blotting were again used to verify that identified colonies retained the plasmid with the insert and that the plasmids were expressing. To identify potential functional domains, both toxins were analyzed using InterPro (*63*).

### Identification of haloarcheal CIS gene clusters and phylogenetic analysis

Representative RefSeq genomes belonging to class *Halobacteria* (halophilic archaea) were downloaded from the National Center for Biotechnology Information (NCBI) (*64*). BLASTP (*65*) was used to search these genomes for gene clusters that potentially encode for CISs, by using Cis2 (*Hbor_38820*) as a query. The whole *Hb* gene cluster was then searched against these filtered genomes to identify the boundaries of each of the detected CIS gene clusters. These gene clusters were then annotated and visualized using clinker 0.0.28 (*66*). 16*S* rDNA sequences and Cis11a protein sequences from each strain were extracted for phylogenetics analysis. In addition, protein sequences encoding for Cis11a found across all bacterial and archaeal genomes were obtained from the eCIStem database (*14*) and were used in the construction of the phylogenetic tree containing bacteria and haloarchaea. Sequence alignments were performed through Clustal Omega 1.2.4 (*67*), and the resulting alignments were subsequently trimmed using TrimAl 1.3 (--automated1) (*68*). FastTree 2.1.11 (*69*) and Interactive Tree of Life (*70*) were used to generate and visualize approximately maximum-likelihood phylogenetic trees using the trimmed alignments as inputs, respectively.

### Bioinformatics analysis of putative membrane anchor

The gene *Hbor_38620* was run through DeepTMHMM to determine if the protein has transmembrane helices and the overall topology of the protein (*71*). The determined transmembrane helices were then identified in the AlphaFold structure and displayed using ChimeraX (*56*, *72*).

### Confocal microscopy of putative membrane protein tagged with GFP

An *Hv* strain expressing the GFP-tagged putative membrane protein (*Hbor_38620*) was grown for 24 hours at 45°C. A 20-μl aliquot of this culture was spotted onto an agarose pad [2.3 M NaCl, 88.3 mM MgCl_2_·6H_2_O, 85.3 mM MgSO_4_·7H_2_O, 56.5 mM KCl, 12 mM tris-HCl pH 7.5, 3 mM CaCl_2,_ and 1% agarose] and left to dry at room temperature. After 30 min, a cover slip was placed onto the sample and then imaged using the Zeiss LSM900 with Airyscan 2 detector and a x63/1.4–numerical aperture oil-immersion objective. *z* stacks of target cells were taken using a confocal imaging track detecting transmitted light and a separate Airyscan track detecting GFP. Collected confocal stacks were deconvolved with the Zeiss LSM Plus processing function, and the Airyscan images were processed using Zeiss deconvolution (jDCV, 10 iterations) in ZenBlue (v.3.5). Minimum-intensity projections of the transmitted light channel and extraction of single Airyscan slices were performed in Fiji (*57*).

### Statistical analysis

Data analysis and statistics mentioned in the text, including SDs and two-way analysis of variance (ANOVA) analysis, were performed in Microsoft Excel and GraphPad Prism.

## Supplementary Materials

### The PDF file includes:

Figs. S1 to S8 Tables S1 to S5 Legends for movies S1 and S2 References
