# Supplementary material for: Archaeal type six secretion system mediates contact-dependent antagonism
Source: Sci Adv. 2024 Nov 15;10(46):eadp7088. doi: 10.1126/sciadv.adp7088 (PMC11566945; doi:10.1126/sciadv.adp7088)
Supplement: Supplementary file 1 — Figs. S1 to S8 Tables S1 to S5 Legends for movies S1 and S2 References [file sciadv.adp7088_sm.pdf]

Science Advances
AAAS

Supplementary Materials for

Archaeal type six secretion system mediates contact-dependent antagonism

Tobias Zachs *et al.*

Corresponding author: Martin Pilhofer, pilhofer@biol.ethz.ch

Sci. Adv. **10**, eadp7088 (2024)
DOI: 10.1126/sciadv.adp7088

The PDF file includes:

Figs. S1 to S8
Tables S1 to S5
Legends for movies S1 and S2
References

Other Supplementary Material for this manuscript includes the following:

Movies S1 and S2

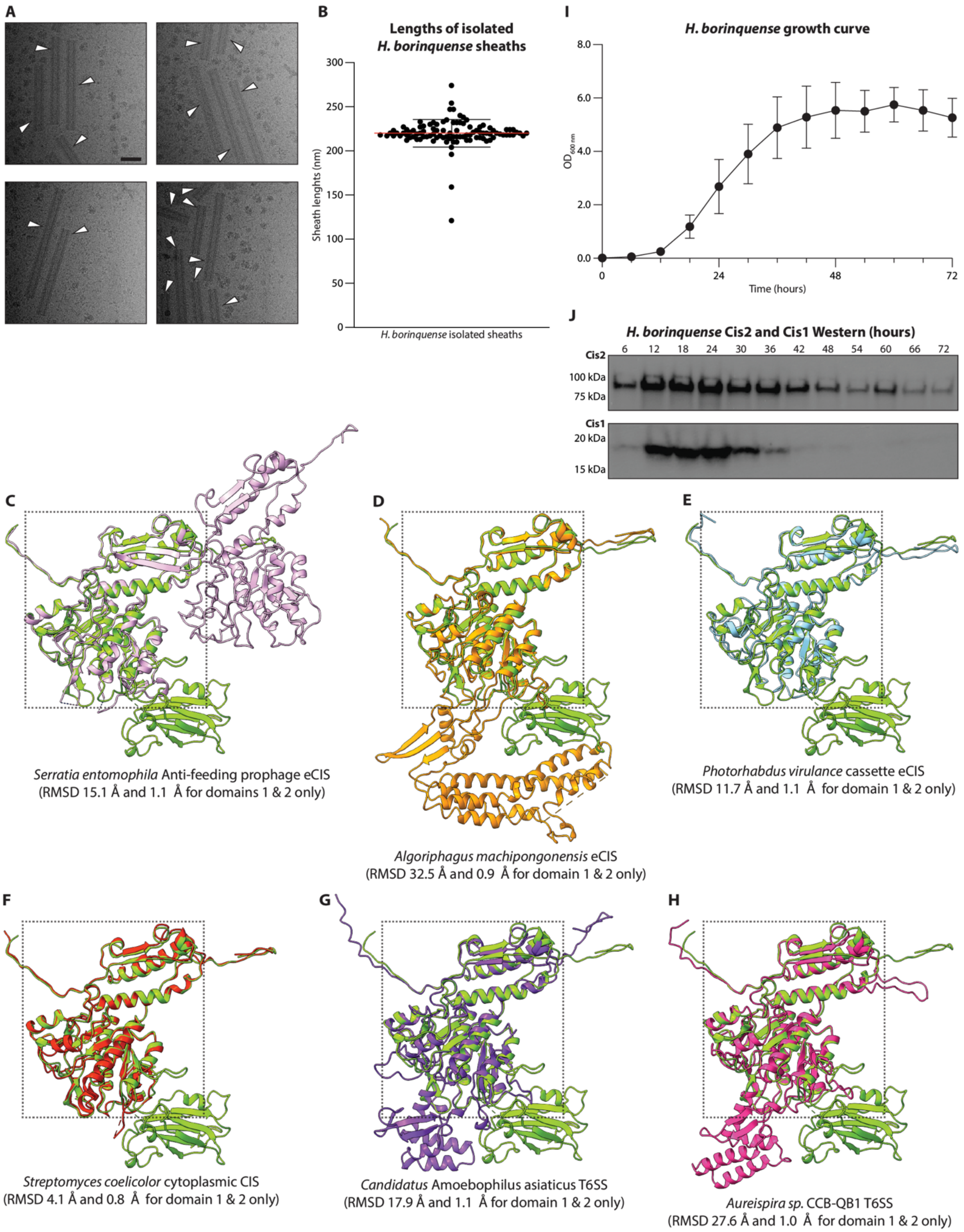

Figure S1: Sheath length distribution, structure comparison and Western blot analysis.

(A) Four example EM micrographs of sheath purifications, revealing length variations of contracted *H. borinquense* sheaths (arrowheads). Scale bar: 50 nm.

(B) Chart showing the length distribution of 100 sheaths from 89 micrographs. A mean length of 220 nm ±16 nm SD was measured, with a maximum length of 274 nm and a minimum length of 121 nm.

(C-H) Alignment of the contracted sheath monomer from *H. borinquense* onto that of *Serratia entomophila* in pink (PDB: 6RC8 (*21*)), *Algoriphagus machipongonensis* in orange (PDB: 7AEK (*24*)), *Photorhabdus* virulence cassette in blue (PDB: 6J0C (*22*)), *Streptomyces coelicolor* in grey (PDB: 8BKY (*28*)), *Candidatus* Amoebophilus asiaticus in purple (AlphaFold of protein Aasi_1074) and *Aureispira* sp. CCB-QB1 in magenta (AlphaFold of protein WP_052593666.1). The structures show high structural similarities in domains 1 and 2 (outlined by the black box, RMSD of 1.0 - 1.7 Å) with large variations in domain 3.

(I) Growth curve of a *H. borinquense* culture initiated at OD 0.01 with a late exponential phase pre-culture. *H. borinquense* was grown in 25 ml YPC at 45˚C and 200 rpm. Points were measured every 6 h over the course of 72 h.

(J) Western blot of *H. borinquense* samples at 6 h intervals. Using antibodies, against the Cis2 (sheath, ~63.3 kDa) and Cis1 (inner tube, ~16.4 kDa), the expression levels of these two proteins was analyzed over 72 h. Samples loaded on the gel were normalized to be at OD 1.0. Expression of sheath remained similar during exponential phase, however, Cis1 expression dropped at mid-exponential phase around 24 h.

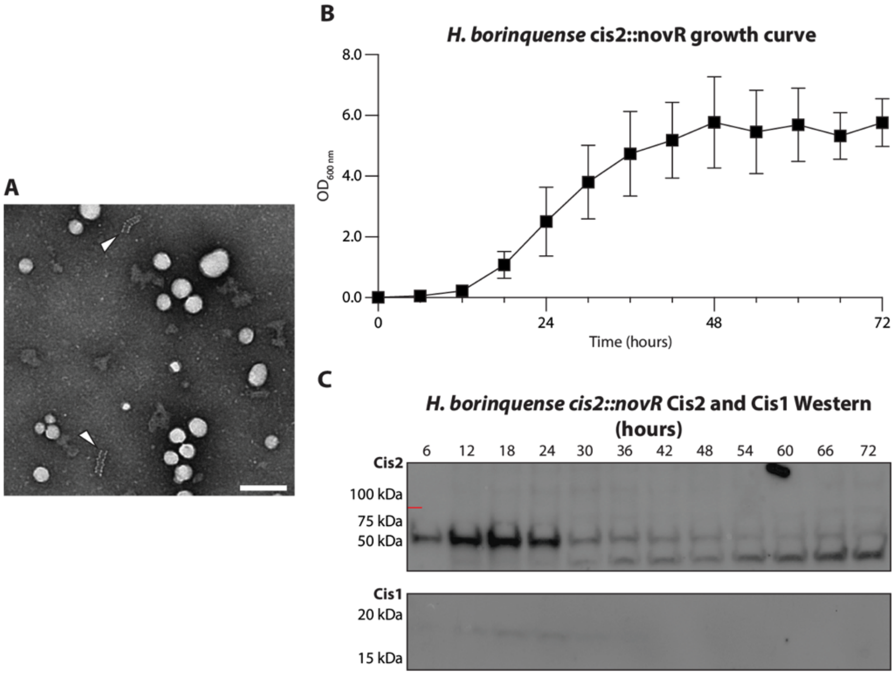

Figure S2: *H. borinquense* c*is2*::*novR* mutant analysis.

(A) Representative negative stain EM micrograph of the sheath isolates from the c*is2*::*novR* mutant *H. borinquense* strain generated by homologous recombination. Few potential sheath-like structures were observable. Identified structures are shorter and look irregular (white arrow). Scale bar: 200 nm.

(B) Growth curve of a *H. borinquense* c*is2*::*novR* culture initiated at OD 0.01 with a late exponential phase pre-culture. The *H. borinquense* sheath mutant was grown in 25 ml YPC at 45˚C and 200 rpm. Points were measured every 6 h over the course of 72 h.

(C) Western blot of *H. borinquense* c*is2*::*novR* growth curve collected at 6 h intervals. Using antibodies against Cis2 (sheath, ~63.3 kDa) and Cis1 (inner tube, ~16.4 kDa), the expression levels of these two proteins was analyzed over 72 h. Samples loaded on the gel were normalized to be at OD 1.0. Bands for the Cis2 correspond to a much shorter protein than that of the full-length Cis2 (red line) and Cis1 expression was barely visible in blots.

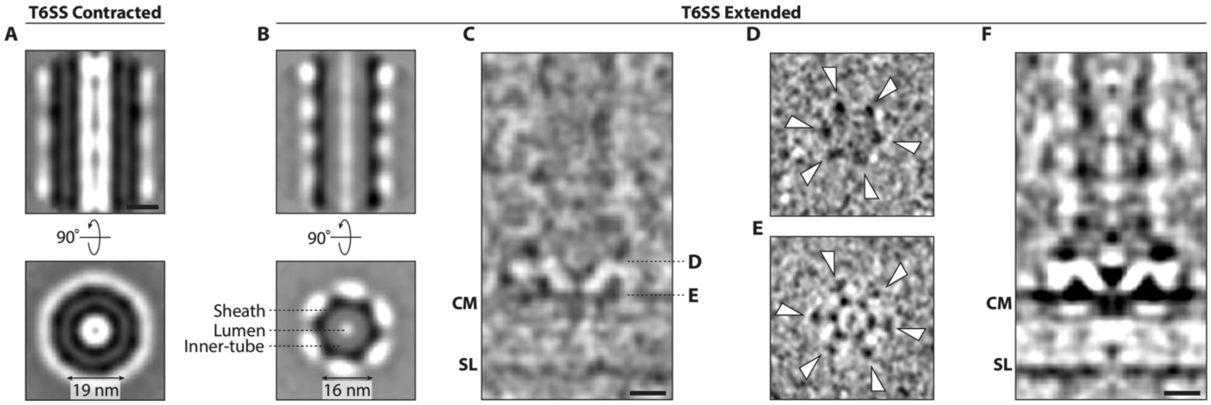

Figure S3: The T6SS is composed of sheath-tube, baseplate and membrane-anchor modules that possess C6 symmetry.

(A) Contracted T6SS sheath subtomogram average. Shown are 1.36 nm thick longitudinal and perpendicular slices through the subtomogram average. In the contracted state, the sheath has a diameter of around 19 nm with no clear density in the center.

(B) Subtomogram average of the T6SS sheath in the extended state. Shown are 1.36 nm thick longitudinal and perpendicular slices through the subtomogram average. In the extended state, the sheath-tube module has a diameter of around 16 nm. A clear density for the sheath is observable with a more diffuse density for the inner tube. In the subtomogram average the lumen of the inner tube appears to be empty.

(C-E) Subtomogram average of the *H. borinquense* T6SS in the extended state without any applied symmetry. The average consists of 57 particles extracted from *H. borinquense* tomograms. Shown are 1.36 nm thick longitudinal and perpendicular slices. In panel **C** all three modules (sheath-tube, baseplate and membrane anchor) of the *H. borinquense* T6SS are visible. Panel **D** is a slice through the baseplate and shows six densities at the edge of the baseplate (arrowheads). Panel **E** is a slice just above the cytoplasmic membrane and shows that there are 13 membrane attachment points. Six contacts are made by both the inner and outer ring ('feet-like' structures highlighted using arrowheads), and a single attachment at the center of structure ('cage'). Scale bar: 10 nm.

(F) Average in panel **C** with an applied C6 rotational symmetry. Scale bar: 10 nm.

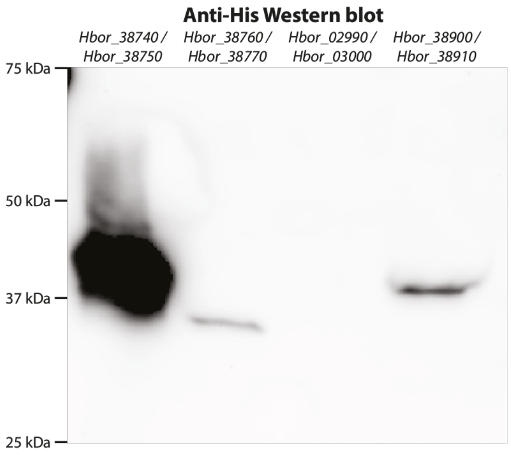

Figure S4: Expression from plasmids expressing toxins with their immunity protein.

Anti-His Western blotting of *H. volcanii* transformed with plasmids expressing identified toxins in tandem with downstream 'immunity' proteins. Expression of three out of four immunity genes (*Hbor*_38750 = 38.9 kDa, *Hbor_38770* = 30.5 kDa and *Hbor_38910* = 35.1 kDa) was detectable. Of note is that halophilic proteins often do not run properly due to their high number of charged residues.

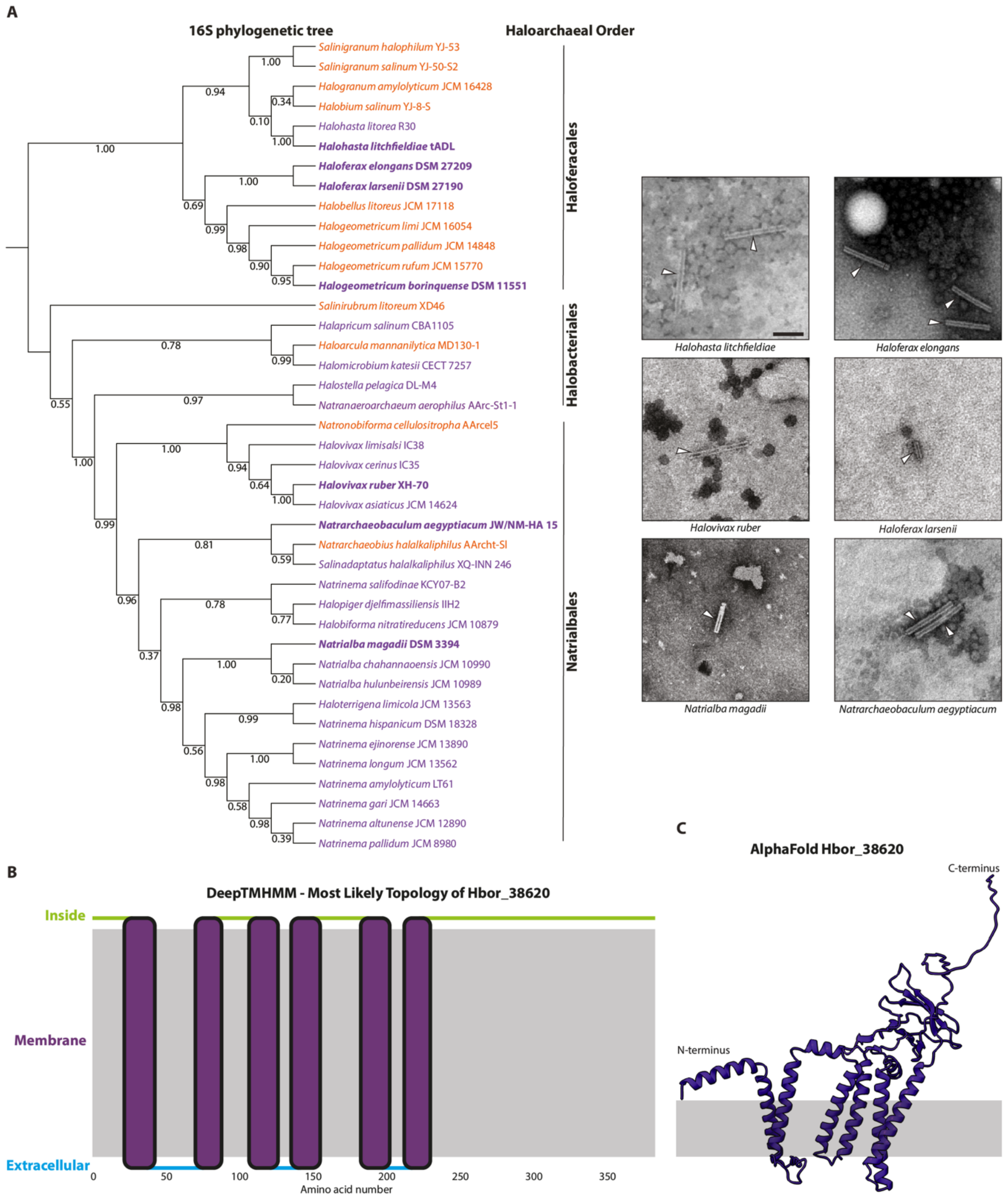

Figure S5: Bioinformatic analysis of haloarchaeal CIS gene clusters reveals conserved putative membrane anchor.

(**A)** 16S rDNA phylogenetic tree of haloarchaeal strains which have a CIS gene cluster. Representatives of three out of four different haloarchaeal orders can be identified. Color coding of the species matches Figure 5A. The mismatch between 16S rDNA and Cis11 phylogenies

indicates horizontal gene transfer of the CIS gene clusters. Example negative stain micrographs of identified sheath structures from six additional haloarchaeal strains. These strains are written in bold within the phylogenetic tree.

(B) Results of the DeepTMHMM transmembrane helices prediction tool indicates that *Hbor_38620* has six transmembrane helices. It also predicts that the putative membrane protein has both the C- and N-terminus localized in the cytoplasm of the cell.

(C) AlphaFold-predicted structure of *Hbor_38620* embedded within the cytoplasmic membrane. The putative membrane protein is predicted to have six transmembrane helices with a highly unstructured C-terminus. We speculate that this C-terminus may facilitate T6SS membrane anchoring by interacting with T6SS baseplate components.

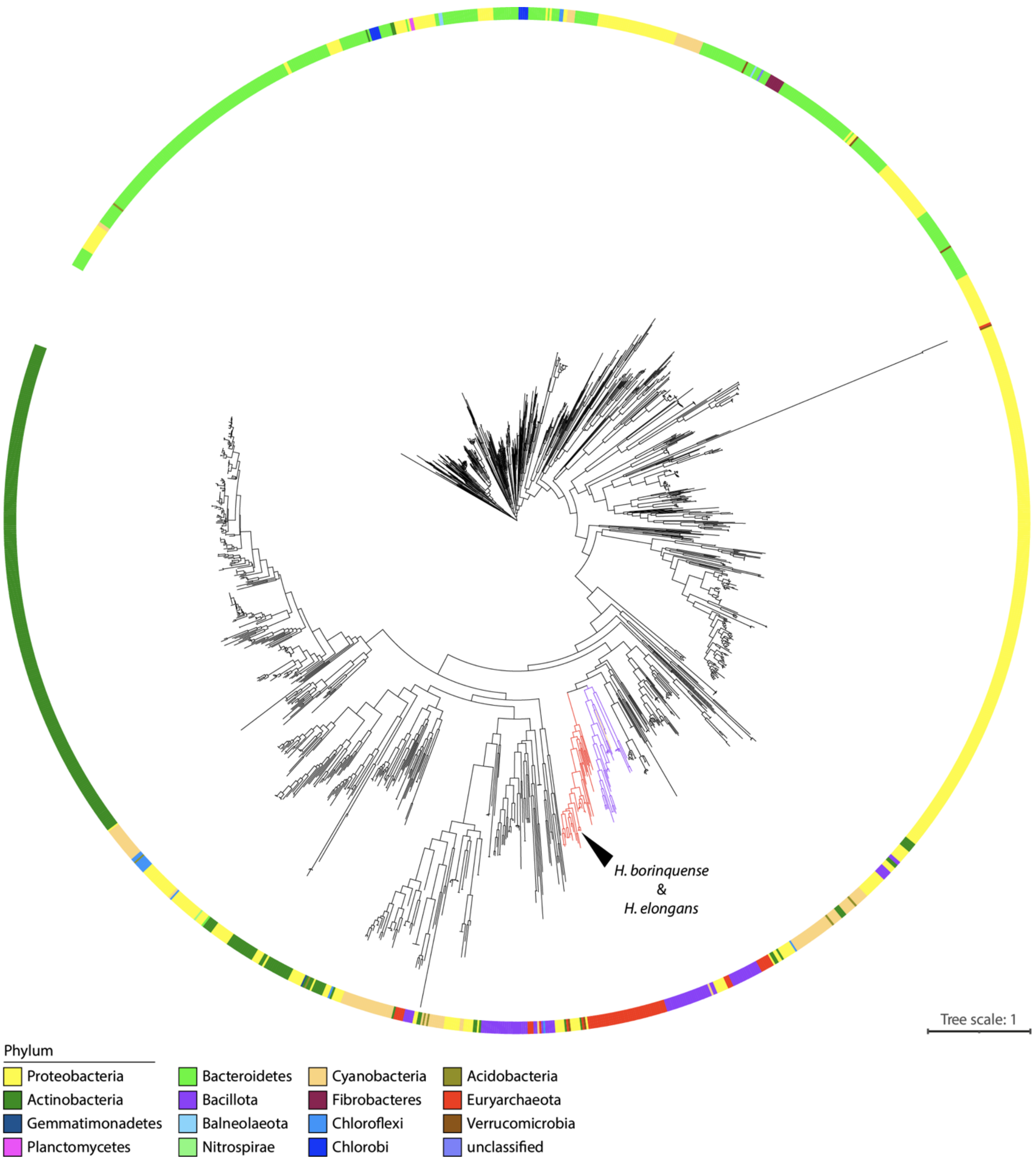

Figure S6: Haloarchaeal CISs cluster with CISs from Bacillota.

Phylogenetic analysis of Cis11 representatives from bacterial and archaeal genomes (similar to a previous study (*14*)). Originating phylum of each sequence is indicated by colors on the outer ring of the tree. Phylogenetic tree branches coming from haloarchaea CIS gene cluster sequences are in red. The branches colored in purple correspond to Gram-positive Bacillota (Firmicutes), which

harbor the closest known relatives to the haloarchaeal CIS. The location of *Halogeometricum borinquense* and *Haloferax elongans* are indicated by the arrowhead.

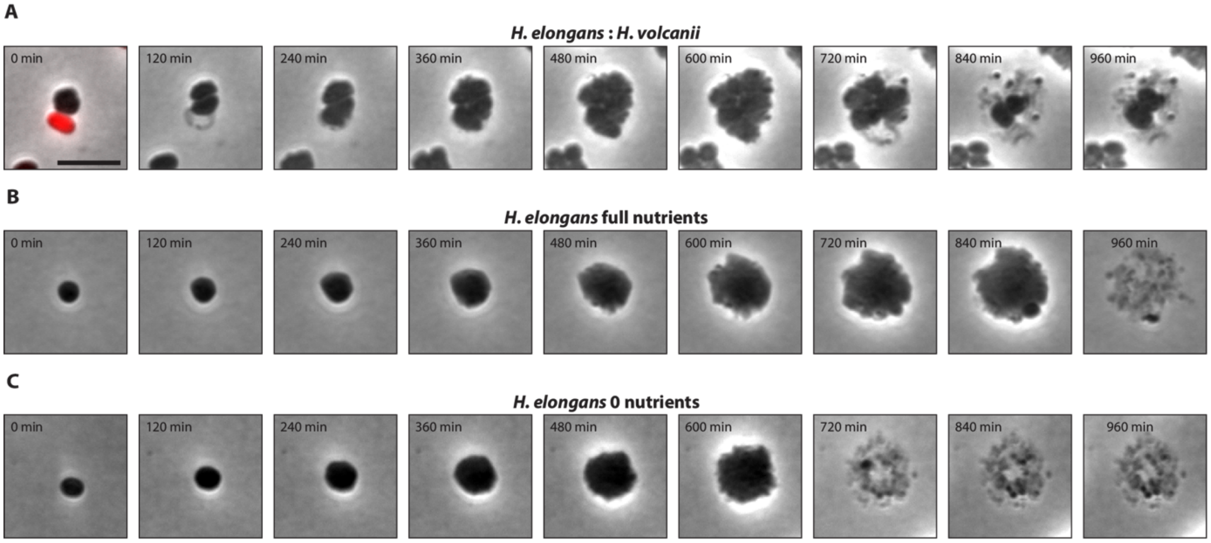

Figure S7: *H. elongans* occasionally undergoes cell lysis during time lapse imaging.

(A-C) Time lapse imaging (15 min frame rate) of *H. elongans* cells that undergo cell lysis while imaged over 960 min at 45˚C. In panel **A**, a selection of frames is shown as overlays of phase contrast images with red fluorescence (respective time points are indicated). Red fluorescence originated from mScarlet expressed in *H. volcanii*. During imaging, *H. volcanii* is observed to undergo a "cell lysis" event at a time point between 0 and 120 min. *H. elongans* is also observed to begin lysing between time point 600 and 960 min. In panels **B** and **C**, a selection of frames are shown, in which *H. elongans* grows in the absence of *H. volcanii*. Both under full and 0 nutrient conditions, *H. elongans* undergoes "cell lysis" before the end of the time lapse imaging. Scale bar: 5 μm.

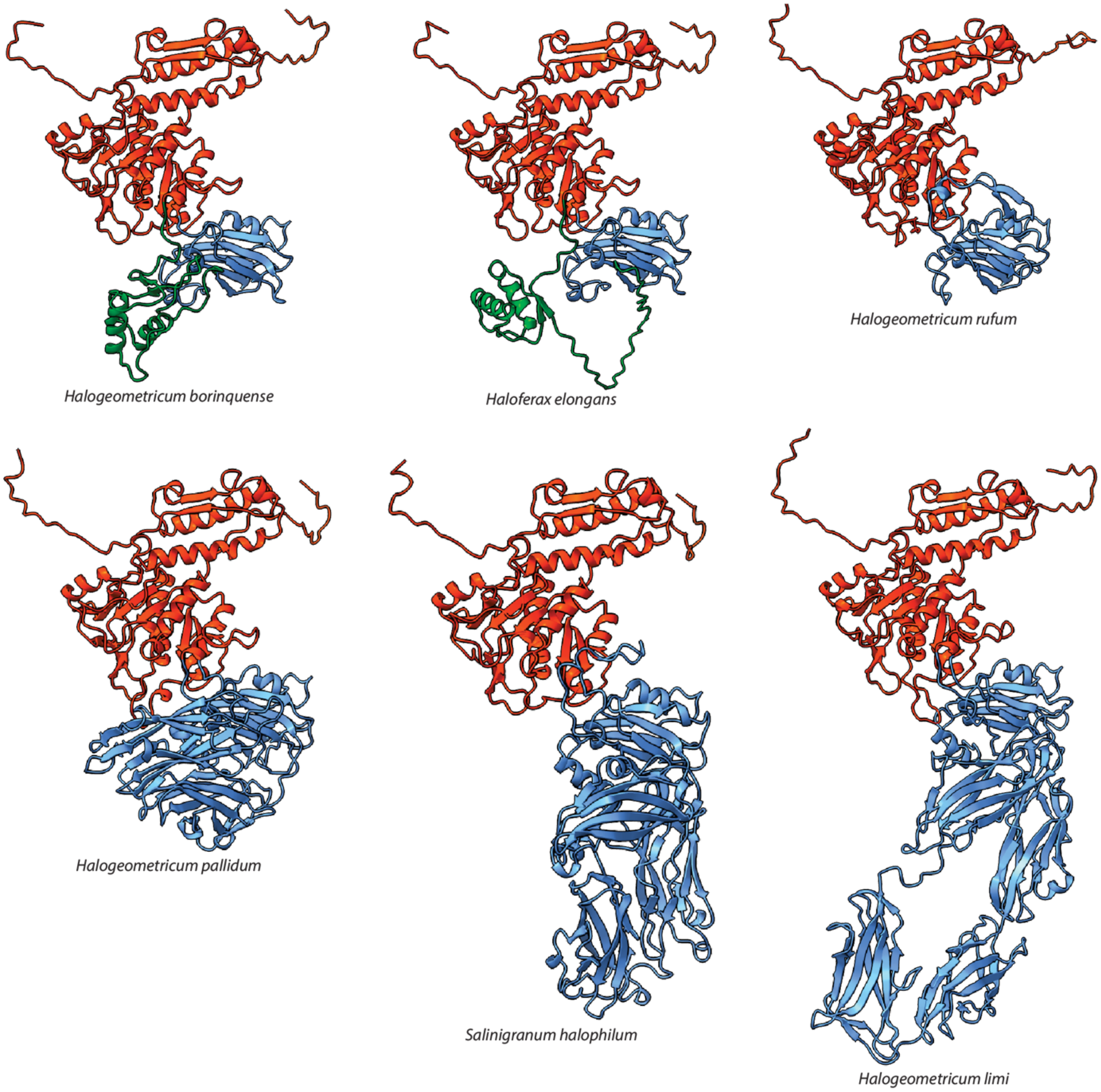

Figure S8: Haloarchaeal Cis2 structure predictions.

Shown are AlphaFold-predicted Cis2 structures from *H. borinquense* and five other haloarchaeal representatives. We validated the accuracy of the predicted structures by comparing the predicted Cis2 structure from *H. borinquense* against the experimentally solved Cis2 structure. With a RMSD of 2.6 Å, these two models are almost indistinguishable. The major difference is the prediction of a fourth domain in the previously unresolved region between domains 1 and 2 (residues 209-291). To assist in comparisons, domains are colored according to the four domains that were identifiable. Domains 1 and 2, in orange, as well as domain 3, in blue, are found in the Alphafold structure of all bioinformatically identified haloarchaeal Cis2 structures. Domain 4, in

green, corresponds to a region in the AlphaFold prediction that was not resolved during the helical reconstruction of *Halogeometricum borinquense* due to the region's high flexibility. Overall, there are three classes of Cis2. The first are those that contain domains 1-4 like *H. borinquense* and *H. elongans* (WP_008322035.1). The second only have domains 1-3 like *H. rufum* (WP_089810112.1). The third do not have domain four and instead have an extension of domain 3 by 2-4 Ig domains (*H. pallidum* (WP_008385220.1)*, S. halophilum* (WP_142860836.1) and *H. limi* (WP_217642716.1).

**Cis component**	**Hypothetical function**	**Gene number**
cis1	Inner tube; in the lumen of the contractile sheath	*Hbor_38830*
cis2	Contractile sheath; facilitating contractile mechanism	*Hbor_38820*
cis5	Inner tube adaptor	*Hbor_38870*
cis7	Baseplate wedge	*Hbor_38640*
cis8a	VgrG spike; at the tip of the contractile structure	*Hbor_38650*
cis8b	VgrG spike; at the tip of the contractile structure	*Hbor_38660*
cis9	Baseplate wedge	*Hbor_38680*
cis10	PAAR; at the tip of VgrG	*Hbor_38670*
cis11a	Baseplate wedge; 'cage' surrounding the tip of the contractile structure	*Hbor_38690*
cis11b	Baseplate wedge; 'cage' surrounding the tip of the contractile structure	*Hbor_38700*
cis12	Baseplate wedge	*Hbor_38710*
cis13	Tail-fiber protein	*Hbor_38810*
cis15	ATPase	*Hbor_38890*
cis16	Cap protein at the distal end of the structure	*Hbor_38880*

Table S1: ***H. borinquense cis*** **components.**

Haloarchaeal strains for CIS identification and functional assays			
Strain name (**source**)	Description	Growth conditions	CIS gene cluster
Halogeometricum borinquense (**DSM 11551**)	wild-type (*Hb*)	45˚C, YPC or DSM 372 or Ca	Yes
Haloferax volcanii (**DSM 3757**)	wild-type (*Hv*)	45˚C, YPC or Ca	No
Haloferax volcanii mScarlet (**mScarlet**)	Strain H26 with mScarlet-I expression plasmid with N-term 6xHis (pSVA5921) (*Hv*-mScarlet)	45˚C, Ca	No
Halogeometricum borinquense sheath mutant (**This study**)	NovR gene homologously recombined into sheath (*Hbor_38820*) in *Hb* (*Hb-cis2*::*novR*)	45˚C, YPC	Yes, disrupted
Haloferax elongans (**DSM 27209**)	wild-type (*He*)	45˚C, YPC	Yes
Haloferax larsenii (**DSM 27190**)	wild-type	45˚C, YPC	Yes
Halogeometricum limi (**JCM 16054**)	wild-type	37˚C, YPC	Yes
Halogeometricum rufum (**JCM 15770**)	wild-type	37˚C, YPC	Yes
Halogeometricum pallidum (**JCM 14848**)	wild-type	37˚C, YPC	Yes
Haloterrigena hispanica (**DSM** 18328)	wild-type	35˚C, DSM 372	Yes
Natrinema pallidum (**DSM 3751**)	wild-type	37˚C, DSM 372	Yes
Halovivax ruber (**DSM 18193**)	wild-type	37˚C, DSM 514 with 10% NaCl	Yes
Halohasta litchfieldiae (**DSM 22187**)	wild-type	28˚C, DSM 1091	Yes
Natrialba magadii (**DSM 3394**)	wild-type	37˚C, DSM 371	Yes
Natrialba chahannaoensis (**DSM 29986**)	wild-type	37˚C, DSM 371	Yes
Natrialba hulunbeirensis (**DSM 29987**)	wild-type	37˚C, DSM 371	Yes
Halomicrobium katesii (**DSM 19301**)	wild-type	37˚C, DSM 1164	Yes
Natrinema ejinorense (**DSM 18194**)	wild-type	37˚C, DSM 1184	Yes
Halopiger djelfimassiliensis (**DSM 27506**)	wild-type	40˚C, DSM 1520	Yes
Halopiger salifodinae (**DSM 26231**)	wild-type	37˚C, DSM 1528	Yes

***Escherichia coli* strains for generating constructs**		
Strain	Description	Source
Top10	*F– mcrA Δ(mrr-hsdRMSmcrBC) Φ80lacZΔM15 ΔlacX74 recA1 araD139 Δ(ara leu) 7697 galU galK rpsL (StrR) endA1 nupG*	Invitrogen
Dam⁻/Dcm⁻ K12	*ara-14 leuB6 fhuA31 lacY1 tsx78 glnV44 galK2 alT22 mcrA dcm-6 hisG4 rfbD1 (zgb210::Tn10) Tet^s endA1*	New England BioLabs

	rspL136 (Str^R) dam13::Tn9 (Cam^R) xylA-5 mtl-1 thi-1 mcrB1 hsdR2	

Table S2: Strains and culturing conditions.

Plasmid (**source)**	Description	Application
pWL102 (**DSM 5717**)	*E. coli - Hv* shuttle vector for selection and maintenance in both bacteria and archaea; ampicillin and lovastatin resistance	Vector used to generate expression plasmids and in functional assays
pWL102et (**This study**)	Plasmid with pWL102 backbone, Pr R promoter (*73*) and 6xHis-tag	For expressing and tagging proteins in *Hv*
pGB68 (*74*)	*E. coli - Hv* shuttle vector for selection and maintenance in both bacteria and archaea; ampicillin and novobiocin resistance	Used to amplify NovR gene
pWL102et NovR (**This study**)	Plasmid with pWL102et backbone and NovR from pGB68	Expression of the antibiotic novobiocin resistance gene
pWL102et int1 (**This study**)	Plasmid with pWL102et backbone and *Hbor_38720* from the *Hb* genome	Expression of interspacing gene 1
pWL102et int2 (**This study**)	Plasmid with pWL102et backbone and *Hbor_38730* from the *Hb* genome	Expression of interspacing gene 2
pWL102et int3 (**This study**)	Plasmid with pWL102et backbone and *Hbor_38740* from the *Hb* genome	Expression of interspacing gene 3
pWL102et int4 (**This study**)	Plasmid with pWL102et backbone and *Hbor_38750* from the *Hb* genome	Expression of interspacing gene 4
pWL102et int5 (**This study**)	Plasmid with pWL102et backbone and *Hbor_38760* from the *Hb* genome	Expression of interspacing gene 5
pWL102et int6 (**This study**)	Plasmid with pWL102et backbone and *Hbor_38770* from the *Hb* genome	Expression of interspacing gene 6
pWL102et int7 (**This study**)	Plasmid with pWL102et backbone and *Hbor_38780* from the *Hb* genome	Expression of interspacing gene 7
pWL102et int8 (**This study**)	Plasmid with pWL102et backbone and *Hbor_38790* from the *Hb* genome	Expression of interspacing gene 8
pWL102et int9 (**This study**)	Plasmid with pWL102et backbone and *Hbor_38800* from the *Hb* genome	Expression of interspacing gene 9
pWL102et int3/4 (**This study**)	Plasmid with pWL102et backbone and *Hbor_38740/38750* from the *Hb* genome	Expression of the immunity/toxin pair 1
pWL102et int5/6 (**This study**)	Plasmid with pWL102et backbone and *Hbor_38760/38770* from the *Hb* genome	Expression of the immunity/toxin pair 2
pWL102et Hbor_01880 (**This study**)	Plasmid with pWL102et backbone and *Hbor_01880* from the *Hb* genome	Expression of the secreted protein *Hbor_01880* without His-tag
pWL102et Hbor_02990 (**This study**)	Plasmid with pWL102et backbone and *Hbor_02990* from the *Hb* genome	Expression of the secreted protein *Hbor_02990* without His-tag
pWL102et Hbor_38720v2 (**This study**)	Plasmid with pWL102et backbone and *Hbor_38720* from the *Hb* genome	Expression of the secreted protein *Hbor_38720* without His-tag
pWL102et Hbor_38900 (**This study**)	Plasmid with pWL102et backbone and *Hbor_38900* from the *Hb* genome	Expression of the secreted protein *Hbor_38900* without His-tag
pWL102et Hbor_02990/03000 (**This study**)	Plasmid with pWL102et backbone and *Hbor_02990/03000* from the *Hb* genome	Expression of the secreted protein *Hbor_02990/03000*

pWL102et Hbor_38900/38910 (**This study**)	Plasmid with pWL102et backbone and *Hbor_38900/38910* from the *Hb* genome	Expression of the secreted protein *Hbor_38900/38910*
pET15b (**Novagen**)	Cloning/expression plasmid with ampicillin resistance	Vector used to make sheath insertion plasmid
pET15b NovR sheath ins (**This study**)	Plasmid with pET15b backbone and sheath insertion fragment	Plasmid for generating *Hb* sheath insertion mutant through homologous recombination
pIDJL40 (*75*)	Plasmid which provided halotolerant GFP	Used to amplify GFP sequence
pWL102et *Hbor_38620-GFP* (**This study**)	Plasmid with pWL102et backbone and *Hbor_38620* from the *Hb* genome with a GFP tag	Expression of putative membrane protein with C-terminal GFP tag

Table S3: Plasmids used in this study.

Plasmid	Name	Sequence (5' to 3')	Purpose
pWL102et	pWL102et_InsertAmp_for	CGGTACCTCTAGAAGAAGCTTGGGATCCA	Amplifying promoter and His-tag
	pWL102et_InsertAmp_rev	GGTCGGACAACAACCCCCCA	Amplifying promoter and His-tag
	pWL102_MCS1_for2	TGGGGGGTTGTTGTCCGACC	Linearizing plasmid pWL102
	pWL102_MCS1_rev2	TGGATCCCAAGCTTCTTCTAGAGGTACCG	Linearized plasmid pWL102
pWL102et NovR	pWL102et_NovR_Amp_for	CAGAAGCCGAACTCTGCACATATGTCTCAGGATAACGAGT	Amplifying NovR from pGB68
	pWL102et_NovR_Amp_rev	TAGTGGTGATGGTGATGATGTTAGATGTCTACCCATTCGG	Amplifying NovR from pGB68
	pWL102et_NT_linear_for	TGGGGGGTTGTTGTCCGACC	Linearized plasmid pWL102et
	pWL102et_CT_linear_rev	ATGTGCAGAGTTCGGCTTCT	Linearized plasmid pWL102et
pWL102et int1	pWL102et_CT_linear_for	CATCATCACCATCACCACTAGT	pWL102et linearizing (for int1-9 expression plasmids)
	pWL102et_CT_linear_rev	ATGTGCAGAGTTCGGCTTCT	pWL102et linearizing (for int1-9 expression plasmids)
	pWL102et_eff1CT_for2	CGAACTCTGCACATATGGGATTTCGGACC	Amplify interspacing region gene 1 (*Hbor_38720*)
	pWL102et_eff1CT_rev	TAGTGGTGATGGTGATGATGTGCATCCCTCCTACTGCTAC	Amplify interspacing region gene 1 (*Hbor_38720*)
pWL102et int2	pWL102et_hypo1CT_for	AGAAGCCGAACTCTGCACATATGGATGGACTAATTACTGG	Amplify interspacing region gene 2 (*Hbor_38730*)
	pWL102et_hypo1CT_rev	TAGTGGTGATGGTGATGATGCTTCGGTTCCCACGACTGGA	Amplify interspacing region gene 2 (*Hbor_38730*)
pWL102et int3	pWL102et_eff2CT_for	AGAAGCCGAACTCTGCACATATGGGAACCGAAGTAGTCCA	Amplify interspacing region gene 3 (*Hbor_38740*)

	pWL102et_eff2CT_rev	TAGTGGTGATGGTGATGATGTGTCAG ACCCCGTGAATCGT	Amplify interspacing region gene 3 (*Hbor_38740*)
pWL102et int4	pWL102et_hypo2CT_for	AGAAGCCGAACTCTGCACATATGAAG GCAAAGATAACCGG	Amplify interspacing region gene 4 (*Hbor_38750*)
	pWL102et_hypo2CT_rev	TAGTGGTGATGGTGATGATGCGTCTC GGTCTGGTCAGTGT	Amplify interspacing region gene 4 (*Hbor_38750*)
pWL102et int5	pWL102et_hypo3CT_for	AGAAGCCGAACTCTGCACATATGCTC CCGCAGACGGGTGC	Amplify interspacing region gene 5 (*Hbor_38760*)
	pWL102et_hypo3CT_rev	TAGTGGTGATGGTGATGATGTTCTCTC ACCCCAGTCGGAT	Amplify interspacing region gene 5 (*Hbor_38760*)
pWL102et int6	pWL102et_hypo4CT_for	AGAAGCCGAACTCTGCACATATGGAC GGAGATATCATCGG	Amplify interspacing region gene 6 (*Hbor_38770*)
	pWL102et_hypo4CT_rev2	TAGTGGTGATGGTGATGATGTTCGTC GGCGAACGCC	Amplify interspacing region gene 6 (*Hbor_38770*)
pWL102et int7	pWL102et_eff3CT_for	AGAAGCCGAACTCTGCACATATGTTT GGCGTGTGGTTCTG	Amplify interspacing region gene 7 (*Hbor_38780*)
	pWL102et_eff3CT_rev	TAGTGGTGATGGTGATGATGGGATTG ATCCGTTCTGATCG	Amplify interspacing region gene 7 (*Hbor_38780*)
pWL102et int8	pWL102et_eff4CT_for	AGAAGCCGAACTCTGCACATATGACC GAGACAGAGCATCC	Amplify interspacing region gene 8 (*Hbor_38790*)
	pWL102et_eff4CT_rev	TAGTGGTGATGGTGATGATGCGCTAG CAAATACGAACCAA	Amplify interspacing region gene 8 (*Hbor_38790*)
pWL102et int9	pWL102et_hypo5CT_for	AGAAGCCGAACTCTGCACATATGAAC GTCACCGAACACTC	Amplify interspacing region gene 9 (*Hbor_38800*)
	pWL102et_hypo5CT_rev	TAGTGGTGATGGTGATGATGTAGATC CGGCCGTGGTGCGA	Amplify interspacing region gene 9 (*Hbor_38800)*
pWL102et int3/4	pWL102et_eff2CT_for	AGAAGCCGAACTCTGCACATATGGGA ACCGAAGTAGTCCA	Amplify interspacing region gene

			3&4 (*Hbor_38740/38750*)
	pWL102et_hypo2CT_rev	TAGTGGTGATGGTGATGATGCGTCTCGGTCTGGTCAGTGT	Amplify interspacing region gene 3&4 (*Hbor_38740/38750*)
pWL102et int5/6	pWL102et_hypo3CT_for	AGAAGCCGAACTCTGCACATATGCTCCCGCAGACGGGTGC	Amplify interspacing region gene 5&6 (*Hbor_38760/38770*)
	pWL102et_hypo4CT_rev2	TAGTGGTGATGGTGATGATGTTCGTCGGCGAACGCC	Amplify interspacing region gene 5&6 (*Hbor_38760/38770*)
pWL102et *Hbor_01880*	pWL102et_Hbor01880_for	GCCGAACTCTGCACATATGGATGGGCTGGATACGAA	Amplify *Hbor_01880*
	pWL102et_Hbor01880_rev	GTGATGATGCATTCATTTGCTCACCTCATCAG	Amplify *Hbor_01880*
pWL102et *Hbor_02990*	pWL102et_Hbor02990_for2	AGAAGCCGAACTCTGCACATATGACTGTCGATACCATG	Amplify *Hbor_02990*
	pWL102et_Hbor02990_rev	TGGTGATGGTGATGATGCATTTATTTTCCTCCTTGGAAGT	Amplify *Hbor_02990*
pWL102et_ *Hbor_07690*	pWL102et_Hbor07690_for	AGAAGCCGAACTCTGCACATATGGGATTTCGATCTACAGA	Amplify *Hbor_07690*
	pWL102et_Hbor07690_rev	GTGATGATGCATTTACTGCCATTGCACCTCAT	Amplify *Hbor_07690*
pWL102et *Hbor_38720 v2*	pWL102et_Hbor38720_for	CGAACTCTGCACATATGGGATTTCGGACCGCACG	Amplify *Hbor_38720*
	pWL102et_Hbor38720_rev	TGGTGATGGTGATGATGCATTTATGCATCCCTCCTACTGC	Amplify *Hbor_38720*
pWL102et *Hbor_38900*	pWL102et_Hbor38900_for2	GCCGAACTCTGCACATATGGCTATCAAGTTTGCA	Amplify *Hbor_38900*
	pWL102et_Hbor38900_rev	TGGTGATGGTGATGATGCATTTAGTTTAGTCCTGAGAACC	Amplify *Hbor_38900*
pWL102et *Hbor_02990/03000*	pWL102et_Hbor02990_for2	AGAAGCCGAACTCTGCACATATGACTGTCGATACCATG	Amplify *Hbor_02990/03000*
	pWL102et_Hbor03000_rev	TAGTGGTGATGGTGATGATGCTTCCCGCCGTAGCCGATGT	Amplify *Hbor_02990/03000*
pWL102et *Hbor_38900/38910*	pWL102et_Hbor38900_for2	GCCGAACTCTGCACATATGGCTATCAAGTTTGCA	Amplify *Hbor_38900/38910*
	pWL102et_Hbor38910_rev	TAGTGGTGATGGTGATGATGTATCGGGGCTGGACGGATGT	Amplify *Hbor_38900/38910*
	SheathInsFragNovR_NovR_for	GGACGGGTACCCGGGGATCTCCC	Amplifying novobiocin

pET15b NovR sheath ins			resistance gene from pGB68
	SheathInsFragNovR_NovR_rev	GTTCTTCTAGATTAGATGTCTACCCATTCGG	Amplifying novobiocin resistance gene from pGB68
	HB_Sheath_for	ATGAGTGAATATCAGTCTCCAG	Amplifying first sheath fragment
	SheathInsFragNovR_sheath1_rev	TATCGGGTACCCGTCCGGCGCGCAGACCATC	Amplifying first sheath fragment
	SheathInsFragNovR_sheath2_for	TCTAATCTAGAAGAACGACATCGACGGTCTG	Amplifying second sheath fragment
	HB_SheathPromoter_rev	TTATGCCTGTTCGGTATCCT	Amplifying second sheath fragment
pWL102et *Hbor_38620-GFP*	pWL102et_Hbor38620_for	CGAACTCTGCACATATGATTCGGCTGTTCTCTAA	Amplifying *Hbor_38620*
	pWL102et_Hbor38620_rev	ATAGAAGAAGAACCACCACCGTCCTGTGGCGATGGGTCT	Amplifying *Hbor_38620*
	pWL102et_sfGFP_for	ACGGTGGTGGTTCTTCTTCTATGAGTAAAGGAGA	Amplifying GFP
	pWL102et_sfGFP_rev	TAGTGGTGATGGTGATGATGTTATTTGTATAGTTCATCCA	Amplifying GFP

Table S4: Oligonucleotides used in this study.

Data collection and processing	
Nominal magnification	81,000
Voltage (kV)	300
Electron exposure (e^-/Å)	~65
Defocus range (μm)	-1 to -3.3
Pixel size (Å/pixel)	1.10
Symmetry imposed	C6 + helical<br>(twist = 33.24°, rise = 17.09 Å)
Final particles (No.)	127,891
Map resolution (Å)	2.9
FSC threshold	0.143
**Refinement**	
Map sharpening B factor ($Å^2$)	-86.8
Model composition	
Non-hydrogen atoms	68,022
Protein residues	8,766
Chains	18
R.M.S deviations	
Bond length (Å)	0.004
Bond angles (°)	0.708
Validation	
MolProbity score	1.09
Clashscore	2.95
Rotamer outlier (%)	0.00
Ramachandran plot	
Favored (%)	98.34
Allowed (%)	1.66
Outlier (%)	0.00
Masked CC	0.87

Table S5: Structure validation of contractile sheath.

Movie S1: *H. volcanii* "cell lysis" event upon *H. borinquense* contact.

Six time lapse imaging examples of *H. volcanii* "lysis" events when in direct contact with *H. borinquense*. Red fluorescence signal is used to distinguish mScarlet-expressing *H. volcanii* from *H. borinquense*. Scale bar: 5 µm.

Movie S2: *H. volcanii* "no proliferation" event upon *H. borinquense* contact.

Six time lapse imaging examples of *H. volcanii* "no proliferation" events when in direct contact with *H. borinquense*. Red fluorescence signal is used to distinguish mScarlet-expressing *H. volcanii* from *H. borinquense*. Scale bar: 5 µm.

REFERENCES AND NOTES

1. M. E. Hibbing, C. Fuqua, M. R. Parsek, S. B. Peterson, Bacterial competition: Surviving and thriving in the microbial jungle. *Nat. Rev. Microbiol.* **8**, 15–25 (2010).

2. C. Moissl-Eichinger, M. Pausan, J. Taffner, G. Berg, C. Bang, R. A. Schmitz, Archaea are interactive components of complex microbiomes. *Trends Microbiol.* **26**, 70–85 (2018).

3. T. R. D. Costa, C. Felisberto-Rodrigues, A. Meir, M. S. Prevost, A. Redzej, M. Trokter, G. Waksman, Secretion systems in Gram-negative bacteria: Structural and mechanistic insights. *Nat. Rev. Microbiol.* **13**, 343–359 (2015).

4. M. van Wolferen, A. Orell, S. V. Albers, Archaeal biofilm formation. *Nat. Rev. Microbiol.* **16**, 699–713 (2018).

5. L. R. Comolli, J. F. Banfield, Inter-species interconnections in acid mine drainage microbial communities. *Front. Microbiol.* **5**, 89594 (2014).

6. S. E. McGlynn, G. L. Chadwick, A. O'Neill, M. Mackey, A. Thor, T. J. Deerinck, M. H. Ellisman, V. J. Orphan, Subgroup characteristics of marine methane-oxidizing ANME-2 archaea and their syntrophic partners as revealed by integrated multimodal analytical microscopy. *Appl. Environ. Microbiol.* **84**, e00399-18 (2018).

7. C. Rudolph, G. Wanner, R. Huber, Natural communities of novel archaea and bacteria growing in cold sulfurous springs with a string-of-pearls-like morphology. *Appl. Environ. Microbiol.* **67**, 2336–2344 (2001).

8. G. Wegener, V. Krukenberg, D. Riedel, H. E. Tegetmeyer, A. Boetius, Intercellular wiring enables electron transfer between methanotrophic archaea and bacteria. *Nature* **526**, 587–590 (2015).

9. A. Besse, J. Peduzzi, S. Rebuffat, A. Carré-Mlouka, Antimicrobial peptides and proteins in the face of extremes: Lessons from archaeocins. *Biochimie* **118**, 344–355 (2015).

10. A. F. Ellen, O. V. Rohulya, F. Fusetti, M. Wagner, S. V. Albers, A. J. M. Driessen, The sulfolobicin genes of Sulfolobus acidocaldarius encode novel antimicrobial proteins. *J. Bacteriol.* **193**, 4380–4387 (2011).

11. K. S. Makarova, Y. I. Wolf, S. Karamycheva, D. Zhang, L. Aravind, E. V. Koonin, Antimicrobial peptides, polymorphic toxins, and self-nonself recognition systems in archaea: An untapped armory for intermicrobial conflicts. *mBio* **10**, e00715-19 (2019).

12. G. L. Chadwick, C. T. Skennerton, R. Laso-Pérez, A. O. Leu, D. R. Speth, H. Yu, C. Morgan-Lang, R. Hatzenpichler, D. Goudeau, R. Malmstrom, W. J. Brazelton, T. Woyke, S. J. Hallam, G. W. Tyson, G. Wegener, A. Boetius, V. J. Orphan, Comparative genomics reveals electron transfer and syntrophic mechanisms differentiating methanotrophic and methanogenic archaea. *PLOS Biol.* **20**, e3001508 (2022).

13. L. Chen, N. Song, B. Liu, N. Zhang, N. F. Alikhan, Z. Zhou, Y. Zhou, S. Zhou, D. Zheng, M. Chen, A. Hapeshi, J. Healey, N. R. Waterfield, J. Yang, G. Yang, Genome-wide identification and characterization of a superfamily of bacterial extracellular contractile injection systems. *Cell Rep.* **29**, 511–521.e2 (2019).

14. A. M. Geller, I. Pollin, D. Zlotkin, A. Danov, N. Nachmias, W. B. Andreopoulos, K. Shemesh, A. Levy, The extracellular contractile injection system is enriched in environmental microbes and associates with numerous toxins. *Nat. Commun.* **12**, 1–15 (2021).

15. M. Brackmann, S. Nazarov, J. Wang, M. Basler, Using force to punch holes: Mechanics of contractile nanomachines. *Trends Cell Biol.* **27**, 623–632 (2017).

16. J. E. Galán, G. Waksman, Protein-injection machines in bacteria. *Cell* **172**, 1306–1318 (2018).

17. N. M. I. Taylor, M. J. van Raaij, P. G. Leiman, Contractile injection systems of bacteriophages and related systems. *Mol. Microbiol.* **108**, 6–15 (2018).

18. N. M. I. Taylor, N. S. Prokhorov, R. C. Guerrero-Ferreira, M. M. Shneider, C. Browning, K. N. Goldie, H. Stahlberg, P. G. Leiman, Structure of the T4 baseplate and its function in triggering sheath contraction. *Nature* **533**, 346–352 (2016).

19. M. Basler, M. Pilhofer, G. P. Henderson, G. J. Jensen, J. J. Mekalanos, Type VI secretion requires a dynamic contractile phage tail-like structure. *Nature* **483**, 182–186 (2012).

20. S. Coulthurst, The type VI secretion system: A versatile bacterial weapon. *Microbiology* **165**, 503–515 (2019).

21. A. Desfosses, H. Venugopal, T. Joshi, J. Felix, M. Jessop, H. Jeong, J. Hyun, J. B. Heymann, M. R. H. Hurst, I. Gutsche, A. K. Mitra, Atomic structures of an entire contractile injection system in both the extended and contracted states. *Nat. Microbiol.* **4**, 1885–1894 (2019).

22. F. Jiang, N. Li, X. Wang, J. Cheng, Y. Huang, Y. Yang, J. Yang, B. Cai, Y. P. Wang, Q. Jin, N. Gao, Cryo-EM structure and assembly of an extracellular contractile injection system. *Cell* **177**, 370–383.e15 (2019).

23. N. J. Shikuma, M. Pilhofer, G. L. Weiss, M. G. Hadfield, G. J. Jensen, D. K. Newman, Marine tubeworm metamorphosis induced by arrays of bacterial phage tail-like structures. *Science* **343**, 529–533 (2014).

24. J. Xu, C. F. Ericson, Y. W. Lien, F. U. N. Rutaganira, F. Eisenstein, M. Feldmüller, N. King, M. Pilhofer, Identification and structure of an extracellular contractile injection system from the marine bacterium *Algoriphagus machipongonensis*. *Nat. Microbiol.* **7**, 397–410 (2022).

25. S. Malfatti, B. J. Tindall, S. Schneider, R. Fähnrich, A. Lapidus, K. LaButtii, A. Copeland, T. G. Del Rio, M. Nolan, F. Chen, S. Lu-cas, H. Tice, J. F. Cheng, D. Bruce, L. Goodwin, S. Pitluck, I. An-derson, A. Pati, N. Ivanova, K. Mavromatis, A. Chen, K. Pa-laniappan, P. D'haeseleer, M. Göker, J. Bristow, J. A. Eisen, V. Markowitz, P. Hugenholtz, N. C. Kyrpides, H. P. Klenk, P. Chain, Complete genome sequence of *Halogeometricum borinquense* type strain (PR3T). *Stand. Genomic Sci.* **1**, 150–158 (2009).

26. R. Montalvo-Rodríguez, R. H. Vreeland, A. Oren, M. Kessel, C. Betancourt, J. López-Garriga, Halogeometricum borinquense gen. nov., sp. nov., a novel halophilic archaeon from Puerto Rico. *Int. J. Syst. Bacteriol.* **48**, 1305–1312 (1998).

27. G. L. Weiss, F. Eisenstein, A. K. Kieninger, J. Xu, H. A. Minas, M. Gerber, M. Feldmüller, I. Maldener, K. Forchhammer, M. Pilhofer, Structure of a thylakoid-anchored contractile injection system in multicellular cyanobacteria. *Nat. Microbiol.* **7**, 386–396 (2022).

28. B. Casu, J. W. Sallmen, S. Schlimpert, M. Pilhofer, Cytoplasmic contractile injection systems mediate cell death in Streptomyces. *Nat. Microbiol.* **8**, 711–726 (2023).

29. D. Böck, J. M. Medeiros, H. F. Tsao, T. Penz, G. L. Weiss, K. Aistleitner, M. Horn, M. Pilhofer, In situ architecture, function, and evolution of a contractile injection system. *Science* **357**, 713–717 (2017).

30. Y.-W. Lien, D. Amendola, K. S. Lee, N. Bartlau, J. Xu, G. Furusawa, M. F. Polz, R. Stocker, G. L. Weiss, M. Pilhofer, Mechanism of bacterial predation via ixotrophy. *Science* **386**, eadp0614 (2024).

31. L. Holm, A. Laiho, P. Törönen, M. Salgado, DALI shines a light on remote homologs: One hundred discoveries. *Protein Sci.* **32**, e4519 (2023).

32. L. Lin, E. Lezan, A. Schmidt, M. Basler, Abundance of bacterial Type VI secretion system components measured by targeted proteomics. *Nat. Commun.* **10**, 2584 (2019).

33. A. Dattani, I. Sharon, E. Shtifman-Segal, S. Robinzon, U. Gophna, T. Allers, N. Altman-Price, Differences in homologous recombination and maintenance of heteropolyploidy between *Haloferax volcanii* and *Haloferax mediterranei*. *G3 (Bethesda)* **13**, jkac306 (2023).

34. T. Rodrigues-Oliveira, A. Belmok, D. Vasconcellos, B. Schuster, C. M. Kyaw, Archaeal S-layers: Overview and current state of the art. *Front. Microbiol.* **8**, 307635 (2017).

35. M. van Wolferen, A. A. Pulschen, B. Baum, S. Gribaldo, S. V. Albers, The cell biology of archaea. *Nat. Microbiol.* **7**, 1744–1755 (2022).

36. J. Alcoforado Diniz, Y. C. Liu, S. J. Coulthurst, Molecular weaponry: Diverse effectors delivered by the Type VI secretion system. *Cell. Microbiol.* **17**, 1742–1751 (2015).

37. D. Quentin, S. Ahmad, P. Shanthamoorthy, J. D. Mougous, J. C. Whitney, S. Raunser, Mechanism of loading and translocation of type VI secretion system effector Tse6. *Nat. Microbiol.* **3**, 1142–1152 (2018).

38. T. E. Wood, S. A. Howard, A. Förster, L. M. Nolan, E. Manoli, N. P. Bullen, H. C. L. Yau, A. Hachani, R. D. Hayward, J. C. Whitney, W. Vollmer, P. S. Freemont, A. Filloux, The *Pseudomonas aeruginosa* T6SS delivers a periplasmic toxin that disrupts bacterial cell morphology. *Cell Rep.* **29**, 187–201.e7 (2019).

39. T. R. Bongiovanni, C. J. Latario, Y. Le Cras, E. Trus, S. Robitaille, K. Swartz, D. Schmidtke, M. Vincent, A. Kosta, J. Orth, F. Stengel, R. Pellarin, E. P. C. Rocha, B. D. Ross, E. Durand, Assembly of a unique membrane complex in type VI secretion systems of Bacteroidota. *Nat. Commun.* **15**, 1–16 (2024).

40. E. Durand, V. S. Nguyen, A. Zoued, L. Logger, G. Péhau-Arnaudet, M. S. Aschtgen, S. Spinelli, A. Desmyter, B. Bardiaux, A. Dujeancourt, A. Roussel, C. Cambillau, E. Cascales, R. Fronzes, Biogenesis and structure of a type VI secretion membrane core complex. *Nature* **523**, 555–560 (2015).

41. C. Rapisarda, Y. Cherrak, R. Kooger, V. Schmidt, R. Pellarin, L. Logger, E. Cascales, M. Pilhofer, E. Durand, R. Fronzes, In situ and high-resolution cryo- EM structure of a bacterial type VI secretion system membrane complex. *EMBO J.* **38**, e100886 (2019).

42. X. Yang, M. Long, X. Shen, Effector–immunity pairs provide the T6SS nanomachine its offensive and defensive capabilities. *Molecules* **23**, 1009 (2018).

43. P. Günther, D. Quentin, S. Ahmad, K. Sachar, C. Gatsogiannis, J. C. Whitney, S. Raunser, Structure of a bacterial Rhs effector exported by the type VI secretion system. *PLOS Pathog.* **18**, e1010182 (2022).

44. M. M. Shneider, S. A. Buth, B. T. Ho, M. Basler, J. J. Mekalanos, P. G. Leiman, PAAR-repeat proteins sharpen and diversify the type VI secretion system spike. *Nature* **500**, 350–353 (2013).

45. T. Allers, H. P. Ngo, M. Mevarech, R. G. Lloyd, Development of additional selectable markers for the halophilic Archaeon *Haloferax volcanii* based on the *leuB* and *trpA* genes. *Appl. Environ. Microbiol.* **70**, 943–953 (2004).

46. D. N. Mastronarde, Automated electron microscope tomography using robust prediction of specimen movements. *J. Struct. Biol.*, **152**, 36–51 (2005); https://doi.org/10.1016/j.jsb.2005.07.007.

47. S. Q. Zheng, E. Palovcak, J. P. Armache, K. A. Verba, Y. Cheng, D. A. Agard, MotionCor2: Anisotropic correction of beam-induced motion for improved cryo-electron microscopy. *Nat. Methods* **14**, 331–332 (2017).

48. K. Zhang, Gctf: Real-time CTF determination and correction. *J. Struct. Biol.* **193**, 1–12 (2016).

49. J. Zivanov, T. Nakane, B. O. Forsberg, D. Kimanius, W. J. H. Hagen, E. Lindahl, S. H. W. Scheres, New tools for automated high-resolution cryo-EM structure determination in RELION-3. *Elife* **7**, e42166 (2018).

50. S. He, S. H. W. Scheres, Helical reconstruction in RELION. *J. Struct. Biol.* **198**, 163–176 (2017).

51. P. B. Rosenthal, R. Henderson, Optimal determination of particle orientation, absolute hand, and contrast loss in single-particle electron cryomicroscopy. *J. Mol. Biol.* **333**, 721–745 (2003).

52. P. Emsley, B. Lohkamp, W. G. Scott, K. Cowtan, Features and development of Coot. *Acta Crystallogr. D Biol. Crystallogr.* **66**, 486–501 (2010).

53. Y. Song, F. Dimaio, R. Y. R. Wang, D. Kim, C. Miles, T. Brunette, J. Thompson, D. Baker, High-resolution comparative modeling with RosettaCM. *Structure* **21**, 1735–1742 (2013).

54. P. D. Adams, P. V. Afonine, G. Bunkóczi, V. B. Chen, I. W. Davis, N. Echols, J. J. Headd, L. W. Hung, G. J. Kapral, R. W. Grosse-Kunstleve, A. J. McCoy, N. W. Moriarty, R. Oeffner, R. J. Read, D. C. Richardson, J. S. Richardson, T. C. Terwilliger, P. H. Zwart, PHENIX: A

comprehensive Python-based system for macromolecular structure solution. *Acta Crystallogr. D Biol. Crystallogr.* **66**, 213–221 (2010).

55. E. F. Pettersen, T. D. Goddard, C. C. Huang, G. S. Couch, D. M. Greenblatt, E. C. Meng, T. E. Ferrin, UCSF Chimera—A visualization system for exploratory research and analysis. *J. Comput. Chem.* **25**, 1605–1612 (2004).

56. E. C. Meng, T. D. Goddard, E. F. Pettersen, G. S. Couch, Z. J. Pearson, J. H. Morris, T. E. Ferrin, UCSF ChimeraX: Tools for structure building and analysis. *Protein Sci.* **32**, e4792 (2023).

57. J. Schindelin, I. Arganda-Carreras, E. Frise, V. Kaynig, M. Longair, T. Pietzsch, S. Preibisch, C. Rueden, S. Saalfeld, B. Schmid, J. Y. Tinevez, D. J. White, V. Hartenstein, K. Eliceiri, P. Tomancak, A. Cardona, Fiji: An open-source platform for biological-image analysis. *Nat. Methods* **9**, 676–682 (2012).

58. T. Zachs, A. Schertel, J. Medeiros, G. L. Weiss, J. Hugener, J. Matos, M. Pilhofer, Fully automated, sequential focused ion beam milling for cryo-electron tomography. *Elife* **9**, e52286 (2020).

59. J. R. Kremer, D. N. Mastronarde, J. R. McIntosh, Computer visualization of three-dimensional image data using IMOD. *J. Struct. Biol.* **116**, 71–76 (1996).

60. D. Tegunov, P. Cramer, Real-time cryo-electron microscopy data preprocessing with Warp. *Nat. Methods* **16**, 1146–1152 (2019).

61. J. E. Heebner, C. Purnell, R. K. Hylton, M. Marsh, M. A. Grillo, M. T. Swulius, Deep learning-based segmentation of cryo-electron tomograms. *J. Vis. Exp.* **189**, e64435 (2022).

62. W. E. Wolski, P. Nanni, J. Grossmann, M. d'Errico, R. Schlapbach, C. Panse, prolfqua: A comprehensive R-package for proteomics differential expression analysis. *J. Proteome Res.* **22**, 1092–1104 (2023).

63. T. Paysan-Lafosse, M. Blum, S. Chuguransky, T. Grego, B. L. Pinto, G. A. Salazar, M. L. Bileschi, P. Bork, A. Bridge, L. Colwell, J. Gough, D. H. Haft, I. Letunić, A. Marchler-Bauer,

H. Mi, D. A. Natale, C. A. Orengo, A. P. Pandurangan, C. Rivoire, C. J. A. Sigrist, I. Sillitoe, N. Thanki, P. D. Thomas, S. C. E. Tosatto, C. H. Wu, A. Bateman, InterPro in 2022. *Nucleic Acids Res.* **51**, D418–D427 (2023).

64. N. A. O'Leary, M. W. Wright, J. R. Brister, S. Ciufo, D. Haddad, R. McVeigh, B. Rajput, B. Robbertse, B. Smith-White, D. Ako-Adjei, A. Astashyn, A. Badretdin, Y. Bao, O. Blinkova, V. Brover, V. Chetvernin, J. Choi, E. Cox, O. Ermolaeva, C. M. Farrell, T. Goldfarb, T. Gupta, D. Haft, E. Hatcher, W. Hlavina, V. S. Joardar, V. K. Kodali, W. Li, D. Maglott, P. Masterson, K. M. McGarvey, M. R. Murphy, K. O'Neill, S. Pujar, S. H. Rangwala, D. Rausch, L. D. Riddick, C. Schoch, A. Shkeda, S. S. Storz, H. Sun, F. Thibaud-Nissen, I. Tolstoy, R. E. Tully, A. R. Vatsan, C. Wallin, D. Webb, W. Wu, M. J. Landrum, A. Kimchi, T. Tatusova, M. DiCuccio, P. Kitts, T. D. Murphy, K. D. Pruitt, Reference sequence (RefSeq) database at NCBI: Current status, taxonomic expansion, and functional annotation. *Nucleic Acids Res.* **44**, D733–D745 (2016).

65. S. F. Altschul, W. Gish, W. Miller, E. W. Myers, D. J. Lipman, Basic local alignment search tool. *J. Mol. Biol.* **215**, 403–410 (1990).

66. C. L. M. Gilchrist, Y. H. Chooi, clinker & clustermap.js: Automatic generation of gene cluster comparison figures. *Bioinformatics* **37**, 2473–2475 (2021).

67. F. Sievers, D. G. Higgins, Clustal Omega for making accurate alignments of many protein sequences. *Protein Sci.* **27**, 135–145 (2018).

68. S. Capella-Gutiérrez, J. M. Silla-Martínez, T. Gabaldón, trimAl: A tool for automated alignment trimming in large-scale phylogenetic analyses. *Bioinformatics* **25**, 1972–1973 (2009).

69. M. N. Price, P. S. Dehal, A. P. Arkin, FastTree 2—Approximately maximum-likelihood trees for large alignments. *PLOS ONE* **5**, e9490 (2010).

70. I. Letunic, P. Bork, Interactive Tree Of Life (iTOL) v5: An online tool for phylogenetic tree display and annotation. *Nucleic Acids Res.* **49**, W293–W296 (2021).

71. J. Hallgren, K. D. Tsirigos, M. Damgaard Pedersen, J. Juan, A. Armenteros, P. Marcatili,H. Nielsen, A. Krogh, O. Winther, DeepTMHMM predicts alpha and beta transmembrane proteins using deep neural networks. *bioRxiv* 2022.04.08.487609 [Preprint] (2022); https://doi.org/10.1101/2022.04.08.487609.

72. J. Jumper, R. Evans, A. Pritzel, T. Green, M. Figurnov, O. Ronneberger, K. Tunyasuvunakool, R. Bates, A. Žídek, A. Potapenko, A. Bridgland, C. Meyer, S. A. A. Kohl, A. J. Ballard, A. Cowie, B. Romera-Paredes, S. Nikolov, R. Jain, J. Adler, T. Back, S. Petersen, D. Reiman, E. Clancy, M. Zielinski, M. Steinegger, M. Pacholska, T. Berghammer, S. Bodenstein, D. Silver, O. Vinyals, A. W. Senior, K. Kavukcuoglu, P. Kohli, D. Hassabis, Highly accurate protein structure prediction with AlphaFold. *Nature* **596**, 583–589 (2021).

73. N. Patenge, A. Haase, H. Bolhuis, D. Oesterhelt, The gene for a halophilic β-galactosidase (bgaH) of Haloferax alicantei as a reporter gene for promoter analyses in Halobacterium salinarum. *Mol. Microbiol.* **36**, 105–113 (2000).

74. G. Bitan-Banin, R. Ortenberg, M. Mevarech, Development of a gene knockout system for the halophilic archaeon *Haloferax volcanii* by use of the pyrE gene. *J. Bacteriol.* **185**, 772–778 (2003).

75. I. G. Duggin, C. H. S. Aylett, J. C. Walsh, K. A. Michie, Q. Wang, L. Turnbull, E. M. Dawson, E. J. Harry, C. B. Whitchurch, L. A. Amos, J. Löwe, CetZ tubulin-like proteins control archaeal cell shape. *Nature* **519**, 362–365 (2015).
